# Network Walking charts transcriptional dynamics of nitrogen signaling by integrating validated and predicted genome-wide interactions

**DOI:** 10.1038/s41467-019-09522-1

**Published:** 2019-04-05

**Authors:** Matthew D. Brooks, Jacopo Cirrone, Angelo V. Pasquino, Jose M. Alvarez, Joseph Swift, Shipra Mittal, Che-Lun Juang, Kranthi Varala, Rodrigo A. Gutiérrez, Gabriel Krouk, Dennis Shasha, Gloria M. Coruzzi

**Affiliations:** 1 Center for Genomics and Systems Biology, Department of Biology, New York University, New York, NY 10003 USA; 2 Courant Institute for Mathematical Sciences, New York University, New York, NY 10012 USA; 3 Horticulture and Landscape Architecture/Center for Plant Biology, Purdue University, West Lafayette, IN 47907 USA; 4 Departamento de Genética Molecular y Microbiología, FONDAP Center for Genome Regulation, Millennium Institute for Integrative Biology, Pontificia Universidad Católica de Chile, Santiago, 8331150 Chile; 5 B&PMP, CNRS, INRA, Université de Montpellier, Montpellier SupAgro, Montpellier, 34060 France

## Abstract

Charting a temporal path in gene networks requires linking early transcription factor (TF)-triggered events to downstream effects. We scale-up a cell-based TF-perturbation assay to identify direct regulated targets of 33 nitrogen (N)-early response TFs encompassing 88% of N-responsive Arabidopsis genes. We uncover a duality where each TF is an inducer and repressor, and in vitro cis-motifs are typically specific to regulation directionality. Validated TF-targets (71,836) are used to refine precision of a time-inferred root network, connecting 145 N-responsive TFs and 311 targets. These data are used to chart network paths from direct TF_1_-regulated targets identified in cells to indirect targets responding only *in planta* via Network Walking. We uncover network paths from TGA1 and CRF4 to direct TF_2_ targets, which in turn regulate 76% and 87% of TF_1_ indirect targets *in planta*, respectively. These results have implications for N-use and the approach can reveal temporal networks for any biological system.

## Abstract (web summary)

Temporal control of transcriptional networks enables organisms to adapt to changing environment. Here, the authors use a scaled-up cell-based assay to identify direct targets of nitrogen-early responsive transcription factors and validate a network path mediating dynamic nitrogen signaling in *Arabidopsis*.

## Introduction

Temporal control of transcriptional networks enables organisms to adapt to a changing environment. Thus, a primary goal of systems biology is to reconstruct the order of transcription factor (TF)–target interactions for the underlying gene regulatory networks (GRNs). To this end, researchers have used de novo network inference to learn GRNs in many organisms spanning microbes, plants, and animals^1–3^. However, a major challenge, especially in higher eukaryotes, is genome-wide validation of the accuracy and predictive power of the resulting GRNs. This is largely due to the lack of methods for rapidly validating the inferred TF–target interactions in vivo^1,4^.

Despite advances in the identification of physical interactions of TFs and targets, there is still relatively little known about which genes are transcriptionally regulated in vivo by a majority of TFs. A recent proliferation of TF–target binding data has emerged from high-throughput in vitro approaches such as DNA affinity purification sequencing (DAP-seq)^5^, protein binding microarrays (PBM)^6^, and yeast-one-hybrid (Y1H)^7^. However, *cis*-binding motifs and target genes identified by these methods fail to account for features present in vivo, such as protein–protein interactions, TF combinations, and chromatin structure. Most importantly, TF–target binding data obtained by in vitro methods or by chromatin immunoprecipitation (ChIP) in vivo do not indicate whether the physical TF–target interaction leads to changes in gene expression. Indeed, ChIP is often a poor predictor of TF regulation^8–10^, and is only a snapshot of the most stable TF-binding events under the conditions and time-point assayed^11–14^.

To complement the TF-DNA binding datasets, there is a need for methods to validate TF–target interactions based on a functional assay that takes into account in vivo context. Perturbation of TFs using knockout or overexpressing transgenic lines to identify regulated targets is standard across microbes and higher eukaryotes^15–17^. However, these in vivo approaches are prohibitively time consuming to scale for most eukaryotes. Additionally, it is not possible to distinguish direct from indirect targets in these systems without additional in vivo TF–target binding information, such as ChIP. Moreover, studies across eukaryotes reveal a poor overlap of TF-bound and TF-regulated targets in vivo^8–10^.

To address the need for TF–target assays based on gene expression, we scale-up the throughput of a cell-based temporal TF perturbation system called TARGET (Transient Assay Reporting Genome-wide Effects of Transcription factors)^18^. This TARGET assay can validate direct TF–target interactions based solely on TF-induced changes in gene expression^18–22^ which overcomes many of the limitations described above. Specifically, the TARGET assay can identify candidate direct TF targets based on gene regulation, is a rapid transient assay, and can be performed on isolated cells from any tissue of interest. Importantly, the TF targets identified by this cell-based assay have also been shown to have in planta relevenace^18–21^. Because cell-based transient expression systems have been developed for many multicellular organisms as a quicker alternative to the creation of stable transgenics and mutants^23–25^, the TARGET approach is broadly applicable. To complement existing genome-wide methods, we apply the TARGET system as a medium-throughput tool to characterize mechanisms of TF action and improve the available gold standards of functional TF–target interactions for use in network inference.

**Fig. 1:**
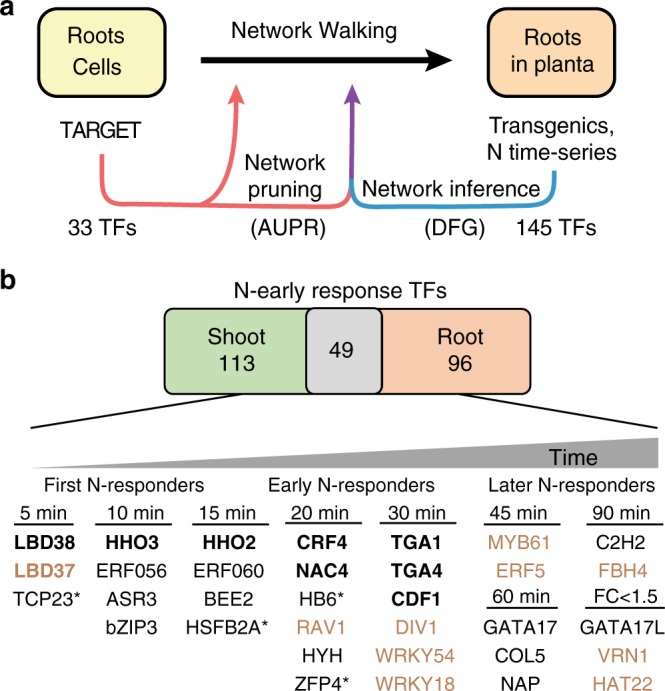
Network Walking connects validated direct transcription factor (TF) targets to in planta responses. **a** Schematic overview: the Network Walking approach charts a network path from direct targets of a TF identified in cells to its indirect targets, which only respond in planta. This is achieved using data for 33 TF perturbations in root cells using TARGET (Transient Assay Reporting Genome-wide Effects of Transcription factors)^18^ scaled-up in this study, and a time-series transcriptome of nitrogen (N) response in whole roots^29^. TF–target edges for 145 TFs were inferred using this time-series data in a machine-learning method called dynamic factor graphs (DFG)^48^ (blue arrow). The inferred edges were pruned for high-confidence edges (purple arrow) using 71,836 validated edges (red arrow) for 33 TFs in a precision/recall analysis (area under precision recall (AUPR)). The validated edges and high-confidence inferred edges are used to link a TF to its indirect targets in planta via the Network Walk. **b** The 33 TFs were selected based on their response to N in shoots and roots (black TFs) or roots only (orange TFs) from the N-treatment time-series data of Varala et al.^29^. TFs were placed into groups based on their Just-in-Time classification, in which genes were binned based on the first time-point N treatment caused a fold change of 1.5 relative to the control^29^. TFs in bold have been previously described in the nitrogen response (CRF4 and CDF1^29^, NAC4^30^, TGA1 and TGA4^26,27^, LBD37 and LBD38^31^, HHO2 and HHO3^32^). Asterisk indicates TFs not included in the DFG network as they did not meet the false discovery rate (FDR) threshold

In this study, we introduce several innovations to scale-up the throughput of the cell-based TARGET system for TF perturbation  to 24 TF assays/day (e.g., 8 TFs × 3 replicates). These innovations have allowed us within ~2 months to identify the candidate direct regulated genome-wide targets of 33 TFs that collectively target ~88% of the genes in the early nitrogen (N) response in *Arabidopsis*. We use this validated TF–target dataset to define a network path that connects the direct targets of these N-early response TFs in root cells to indirect targets identified only in planta. To do this, we present a Network Walking approach that combines functionally validated (85,144 edges) and time-inferred TF–target edges to connect TF targets validated in root cells, with indirect targets regulated in planta, as shown in Fig. 1a. In our proof-of-concept Network Walking examples, we determine the network path for two known TFs in the N response, TGA1^26–28^ and CRF4^29^. Using this approach, we connect 77% and 87% of the indirect targets detected only in planta, back to TGA1 and CRF4, respectively, through intermediate TF_2_s. The Network Walking approach has general application across biological systems. Our proof-of-concept examples have implications for manipulation of networks that control plant N-use efficiency, a process that impacts agriculture, the environment, and human health.

## Results

### Direct regulated targets of 33 N-early response TFs

We sought to identify which of the ~2000 TFs in *Arabidopsis* facilitate the rapid response to N signaling in plant roots, as well as the temporal regulatory paths they employ. To do this, we targeted a set of 33 N-early response TFs for functional testing (Fig. 1b), selected based on their rapid transcriptional response to N treatment in a fine-scale time-course study conducted by Varala et al.^29^. In that study, genes responding to N treatment as function of time (N × Time) were identified by fitting a cubic spline model^29^. These N × Time-responsive genes include 145 TFs in roots and 162 TFs in shoots, with an overlap of 49 TFs (Fig. 1b). We selected a subset of 33 TFs that respond across N × Time in both shoots and roots, or specific to roots, to validate their N-early response network. This set of 33 TFs includes 9 TFs previously validated in the N response (e.g., CRF4 and CDF1^29^, NAC4^30^, TGA1 and TGA4^26–28^, LBD37 and LBD38^31^, HHO2, and HHO3^32^) and 24 TFs with an as yet unknown role in N signaling. We note that this selection approach misses TFs that only respond post-translationally to the N signal, such as NLP7^9,33^, a well-known master regulator of the N response in *Arabidopsis*.

To determine the genome-wide targets regulated by these 33 N-early response TFs, we used the cell-based TARGET system for inducible TF perturbation in root cells^18^ with our modifications to increase throughput. In the TARGET system, TF nuclear entry is controlled using a subdomain of the glucocorticoid receptor (GR) fused to the TF of interest, an approach that has also been used in planta^34,35^. The GR–TF fusion protein is held in the cytoplasm by HSP90-GR binding, and dexamethasone (DEX) treatment disrupts this interaction, allowing temporal control of TF entry into the nucleus^35^. As has been shown in planta^35^ and in isolated roots cells^18^, pre-treatment with cycloheximide (CHX) blocks downstream regulation of secondary TF targets. Thus, candidate direct TF targets can be identified as those that respond transcriptionally to DEX-induced TF nuclear import in the presence of CHX^18,35^.

**Fig. 2:**
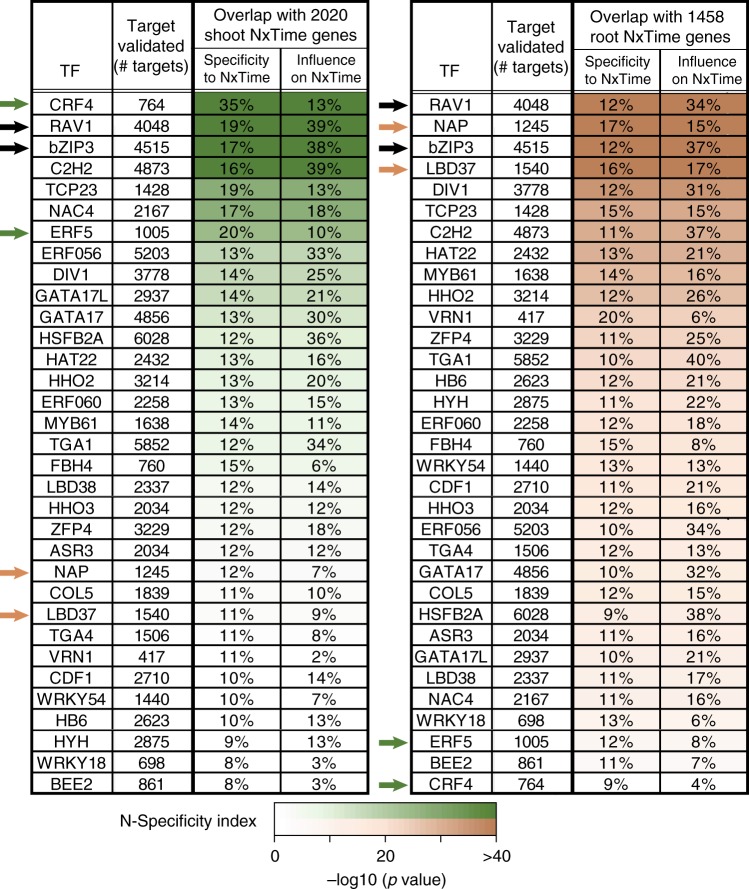
Validated direct targets of the 33 nitrogen (N)-early response transcription factors (TFs) are enriched in N × Time genes. The intersection of direct regulated targets for the 33 N-early response TFs identified in root cells using the TARGET (Transient Assay Reporting Genome-wide Effects of Transcription factors) system with N × Time genes from Varala et al.^29^. This allowed identification of TFs regulating a significant portion of the N response in both roots and shoots (e.g., bZIP3/RAV1, black arrows). The direct regulated targets of other TFs are enriched in organ-specific N × Time response genes. These include CRF4/ERF5, which are specifically enriched for the shoot N × Time response genes (green arrows), and NAP/LBD37, which are specifically enriched for the root N × Time response genes (orange arrows). Green and orange shading represents the N specificity Index^29^, the *p* value calculated using the one proportion *z*-test (see Methods)

In our study, we made two innovations that increased the throughput of the TARGET assay for TF perturbation: (i) the use of an empty vector (EV) control, and (ii) pooling of cells separately transfected with vectors containing either red fluorescent protein (RFP) or green fluorescent protein (GFP), prior to fluorescence-activated cell sorting (FACS) selection of positively transfected cells (see Methods and Supplementary Fig. 1a). Both changes enabled us to increase the throughput of TF perturbations screened via TARGET up to 24 TF assays/day (e.g. 8 TFs × 3 replicates). Additionally, in this design, because all samples are treated with CHX (e.g., TFs and EV), it circumvents the need to compare ±CHX samples, which may impact gene expression and the ability to identify TF-regulated genes (also see Supplementary Fig. 2, Supplementary Data 1, and Supplementary Methods). Using this enhanced medium-throughput TARGET approach, we could identify direct regulated targets of 33 TFs within ~2 months (Fig. 2). To obtain a list of genes differentially expressed (DE) in response to TF perturbation, we performed RNA-seq on root cells collected 3 h after DEX-induced nuclear entry of the GR–TF fusion. We then compared the transcriptome for each of the 33 TF samples (performed in triplicate) to the EV-negative control using the bioinformatics analysis pipeline shown in Supplementary Fig. 1b. The TF targets identified as DE between each of the 33 TFs and EV control (false discovery rate (FDR) < 0.05) are reported in Supplementary Data 2 and represent 85,144 TF-target interactions. Further details on the treatments can be found in Supplementary Figs. 3 and 4.

The number of candidate direct regulated targets for each of the 33 N-early response TFs identified in our enhanced TARGET approach ranged between a low of 417 DE genes (VRN1) to a high of 6028 DE genes (HSFB2A) (Fig. 2). This range in the number of TF targets was not related to differences in TF overexpression level, as there was no correlation between the level of TF expression (compared to EV baseline) and the number of TF-regulated targets (Supplementary Fig. 5). Additionally, we found that there are typically fewer direct regulated targets for a TF detected using TARGET, an in vivo assay, compared to the number of TF-bound targets identified in vitro by DAP-seq^5^ (Supplementary Fig. 6). Indeed, we show that our TARGET data can be used to refine in vitro TF-DNA binding edges to identify which TF–target interactions are likely to result in gene regulation within a plant cell. We found that only a fraction of TF-bound targets from DAP-seq^5^ are regulated by the TF in our root protoplast TARGET assay (Supplementary Fig. 6). However, we did uncover a significant enrichment of direct TF-regulated targets that are also TF-bound in vitro for 13/17 TFs with DAP-seq data^5^ (Supplementary Data 3).

To assess how direct regulated TF targets identified compare with TF targets identified in planta, we examined available in planta ChIP binding data for three TFs, WRKY18^36^, HB6, and HAT22^37^, and found large and significant overlap in each case, despite different experimental growth and treatment conditions (Table 1). Lastly, for TGA1, a well-studied TF in N signaling^26–28^, we observed a large and highly significant overlap (600 genes, *p* value = 1.78E−19, Fisher’s exact test) of direct regulated targets identified in root cells using the TARGET system, compared to DE genes resulting from TGA1 overexpression in roots of whole plants (Supplementary Data 4 and Supplementary Fig. 7) (see Methods). These results collectively support that the candidate direct TF targets identified using the TARGET assay in root cells are enriched in bona fide targets with in planta relevance.

**Table 1:** Direct regulated TF targets from cells significantly overlap with in planta TF binding

TF	Direct regulated targets in root cells (TARGET, this study)	In planta-bound targets (ChIP^36,37^)	No. of genes in overlap	% of genes in overlap	*p* value (Fisher’s exact test)
HAT22	2432	5902	1035/2432	43%	5.6e−56
HB6	2623	7503	1270/2623	48%	3.1e−42
WRKY18	698	805	159/698	23%	3.8e−78

*TF* transcription factor, *TARGET* Transient Assay Reporting Genome-wide Effects of Transcription factors, *ChIP* chromatin immunoprecipitation

### N × Time genes enriched in direct regulated targets of 33 TFs

The 33 N-early response TFs were selected based on their N × Time response in shoots and roots, or roots only, from the study by Varala et al.^29^ (Fig. 1b). We therefore examined whether the direct regulated targets of each TF identified in TARGET overlapped with N × Time-responsive genes in shoots or roots of whole plants. To do this, we calculated the N-response specificity for each TF by determining the percent of the target genes for a TF that are also N × Time-responsive genes in roots or shoots of whole plants (Fig. 2, see Methods). We also determined the influence of each TF on the N × Time genes (i.e., the percent of N × Time genes regulated by a particular TF) for each organ (Fig. 2, see Methods). The 33 TFs in Fig. 2 were ranked based on the N specificity Index^29^ of their validated targets (shown by color shading), a measure of the significance of the influence a TF has on the N × Time genes in each organ (see Methods). Overall, the targets of each of the 33 TFs significantly overlapped with the N × Time genes in shoots and/or roots of whole plants (Fig. 2). However, the ranking of TFs was organ specific. For instance, CRF4, a known TF in the N response in shoots^29^, and ERF5 (Fig. 2, green arrows) are examples of TFs whose direct regulated targets showed organ specificity for the shoot N × Time genes. Conversely, the targets of NAP and the known N-response regulator LBD37^31^ (Fig. 2, orange arrows) showed specificity for root N × Time genes (Fig. 2, brown arrows). Finally, TFs that our study now implicates in the N response, e.g., bZIP3 and RAV1, controlled a significant number of genes that respond to N treatment in both shoots and roots (Fig. 2, black arrows).

### TF–target edges validate a network regulating N processes

**Fig. 3:**
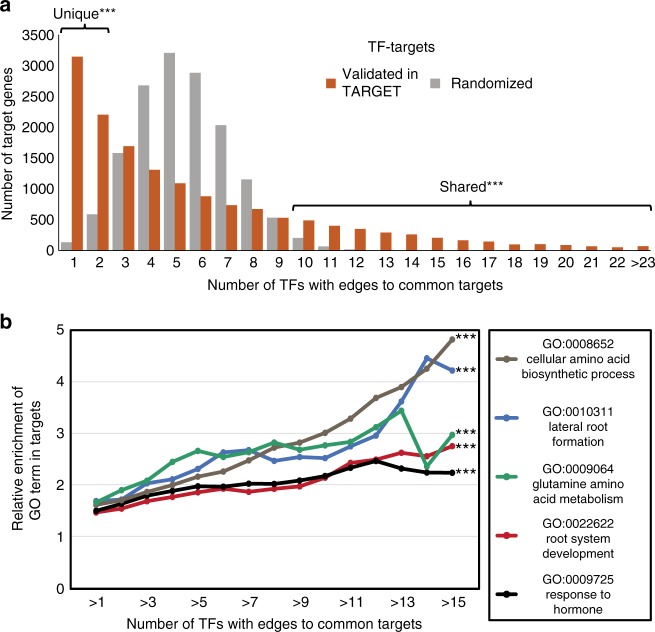
Nitrogen (N)-early response transcription factors (TFs) coordinate a connected network of N-related genes. **a** TF–target edges identified by TARGET (Transient Assay Reporting Genome-wide Effects of Transcription factors) in root cells for the 33 N-early response TFs were used to construct a validated network (orange bars). This validated TF–target network resembles a scale-free distribution, with significantly more unique targets (edges to 1 or 2 TFs) or shared targets (edges to 10 or more TFs), compared to a network which contains the same TFs and targets but with randomized edges (gray bars) (*n* = 1000). **b** Enrichment of gene ontology (GO) terms for N-related processes increases as the number of TFs regulating the set of target genes increases. Significance was tested by calculating a figure of merit, called Focus, for the validated TF–target network and comparing it to the Focus values of randomized networks (*n* = 1000) in which the edges within the validated network were shuffled. (***p value *<* 0.001, permutation test) (see Methods). Source data of Figs. 3a and 3b are provided as a Source Data file

The above TARGET results ranked the 33 N-early response TFs according to their individual roles in regulating genes in the N responses in planta (Fig. 2). We next asked how this set of TFs work together in mediating the N response. Collectively, these 33 TFs regulate 88% of the N × Time geneset in roots (1288/1458 genes; *p* value = 1.55E−67, Fisher’s exact test), and 88% of the N × Time geneset in shoots (1785/2020; *p* value = 5.65E−45 Fisher’s exact test) (Supplementary Table 1). To gain further insight into their collective influence, we explored the network topology for the 33 TFs and their genome-wide targets. To do this, we compared the distribution of TF–target edges within the validated TARGET network for the 33 TFs (Fig. 3a, orange bars), to a network that contains the same TFs and targets but with randomized edges (Fig. 3a, gray bars). The distribution of edges in the validated TARGET network differs significantly from the randomized network. Specifically, compared to the random network, the validated TF–target network contains significantly (*p* value < 0.001, permutation test) more targets that were unique (targeted by only one or two of the TFs) as well as shared (targeted by ≥10 TFs) (Fig. 3a).

We next asked whether the shared targets of the 33 N-early response TFs are enriched for N-related processes. To test this, we calculated the enrichment for Gene Ontology (GO) terms (see Methods). This analysis showed that the collective targets of the 33 TFs were enriched in GO terms such as: N compound metabolic process, cellular amino acid biosynthesis, lateral root formation, and response to hormone (Supplementary Data 5). Additionally, enrichment of N-related GO terms increased as the number of TFs with edges to a set of common targets increased (Fig. 3b). To test whether this increased enrichment is significant, we devised a figure of merit, which we call Focus (see Methods). The Focus for a TF–target network is greater with respect to set of genes (e.g., GO term) when TFs have more edges to that set of genes. This test determines the probability that the Focus calculated from the edges in the TARGET validated network is higher than we would expect to see by chance. For the network of all validated targets of the N-early response TFs, the Focus for each of the GO terms was significantly greater than for the randomized networks (*p* value < 0.001, permutation test) (Fig. 3b).

We also examined how each of these 33 TFs influence the expression of genes involved in N use, including N uptake and assimilation, by plotting a heatmap of the effect each TF had on the expression of these genes (Supplementary Fig. 8). Overall, this set of 33 TFs regulated genes involved in N use more at the level of N reduction and assimilation, compared to N uptake/transport (Supplementary Fig. 8). This finding is consistent with the high enrichment of the cellular amino acid biosynthesis GO term in the shared targets of the 33 TFs (Fig. 3b). These TARGET results can also help define which edges detected from in vitro TF-DNA binding experiments may lead to functional gene regulation. Specifically, our results suggest that TF target binding studies may underestimate (Y1H^28^), or overestimate (DAP-Seq^5^), TF–target interactions within the N-metabolism network, in comparison to the functionally regulated TF targets we identified in root cells using the TARGET assay (Supplementary Fig. 9).

### *Cis*-motifs for a TF are linked to induction or repression

**Fig. 4:**
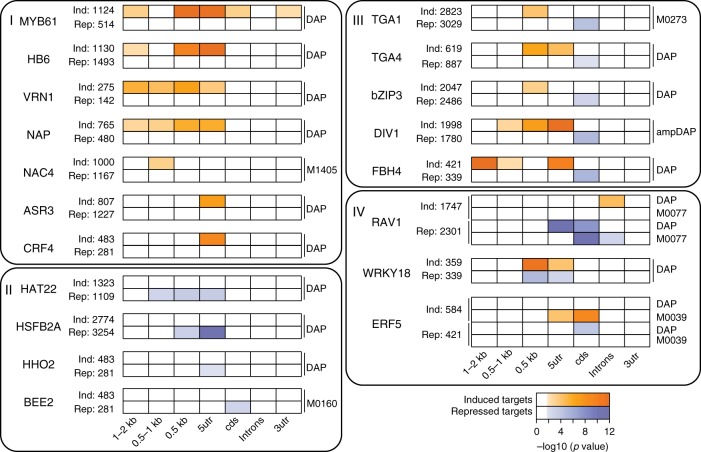
*Cis*-motif enrichment in distinct genic regions is associated with transcription factor (TF) induction or TF repression. TF perturbation assays of the 33 nitrogen (N)-early response TFs in root cells reveals that each TF can act both as an inducer and a repressor of direct target genes (number of targets indicated to the right of TF name). Enrichment analysis revealed that TF *cis*-binding motifs are often specific to a particular type of regulation (e.g., induction vs. repression). *Cis*-motif data available for 21/33 TFs enabled us to classify these TFs into four groups. Targets identified by the cell-based TARGET (Transient Assay Reporting Genome-wide Effects of Transcription factors) assay uncovered TFs whose known *cis*-motifs are associated with induction of targets (group I) and TFs with *cis*-motifs associated with repression of targets (group II). Another group of TFs (group III), including all the bZIP family members in this study, showed *cis*-motif enrichment in the promoter or 5’ untranslated region (UTR) of induced targets, while repressed targets only were enriched for the *cis*-motif in the coding sequence (CDS). The final set of TFs (group IV) showed enrichment of their known *cis*-motifs in the overlapping regions of both induced and repressed targets. HHO3 and TCP23 showed no enrichment of their *cis*-motifs in any region and are not shown. One or two representative motifs for each TF from the following sources are shown: DNA affinity purification sequencing (DAP-seq)^5^ with genomic DNA (DAP), DAP-seq with PCR-amplified gDNA (ampDAP), and Cis-BP^38^ (labeled with motif ID). Orange shading (induced targets) and blue shading (repressed targets) represents the enrichment of motifs as calculated using the AME tool within the MEME suite^69^, with *p* values determined using Fisher's exact test. Results for all *cis*-motifs can be found in Supplementary Data 6

We next used our TARGET data on the direct regulated targets of the 33 N-early response TFs to filter the in vitro TF-DNA binding data for functional regulation in vivo. Notably, each of the 33 TFs acted as both an inducer or as a repressor of distinct sets of target genes (Fig. 4 and Supplementary Data 2). Because of this duality of TF function, we were able to classify known *cis*-binding motifs based on their association with the direction of gene regulation, e.g., induction or repression. We performed this analysis for the 21/33 TFs that have *cis*-binding motif data from DAP-seq^5^ (34 *cis*-motifs), Cis-BP^38^ (16 *cis*-motifs), PBM^39^ (3 *cis*-motifs), or in vivo ChIP^37^ (4 *cis*-motifs). We searched for enrichment of these known *cis*-motifs for each TF in distinct gene regions of induced or repressed direct regulated targets for each TF (Fig. 4, see Methods). For 19/21 TFs and 50/57 *cis*-motifs, we found a significant enrichment (FDR < 0.05, Fisher’s exact test) of at least one *cis*-motif in at least one gene region in the direct TF-regulated targets. When we used all regulated TF targets combined (e.g., induced and repressed), only 15/21 TFs in our study and 40/57 unique *cis*-motifs showed *cis*-motif enrichment (Supplementary Data 6).

Typically, we detected enrichment of known *cis*-motifs for each TF in the proximal promoter or 5’ untranslated region (UTR) (Fig. 4). However, for some *cis*-motifs, we also found enrichment in other gene regions such as the coding sequence (CDS), introns, or 3’UTR (Fig. 4), which has been previously reported^5,40^. Unexpectedly, for 11/21 TFs, their known *cis*-motif was enriched exclusively in either induced TF targets (group I) or repressed TF targets (group II) (Fig. 4). Another unexpected finding was that a set of 5 TFs (group III) showed enrichment of their *cis*-binding motif in the promoter and 5’UTR for induced targets; however, for repressed targets, the *cis*-motif was enriched only in the CDS (Fig. 4). Lastly, three transcription factors, RAV1, WRKY18, and ERF5 (Group IV), displayed enrichment of their *cis*-binding motifs in the same overlapping region for both induced and repressed targets (Fig. 4). We note that the results of our *cis*-binding motif enrichment analysis were similar when we first filtered for accessible chromatin in the 500 bp promoter, as determined in *Arabidopsis* roots by  DNase I hypersensitivity^40^ (Supplementary Data 7). Additionally, the above *cis*-motif enrichment results were also supported when in vitro TF binding (DAP-seq^5^) are intersected with the induced and repressed direct regulated TF targets (Supplementary Data 3).

We next addressed whether the direct regulated TF targets that are not enriched in a known *cis*-binding motif for that TF physically associate with the TF in vivo. To do this, we intersected the induced and repressed regulated targets with in planta-bound targets identified using available ChIP-seq data for HB6 and HAT22^37^ (Supplementary Table 2). For both of these TFs, the induced and repressed direct regulated TF targets overlapped significantly with the in planta TF-bound targets (Supplementary Table 2). This result suggests that either there is an as yet unidentified secondary *cis*-motif or that the TF binds to the target via TF–TF interactions. The latter interpretation is supported by the identification of TF partner elements described below.

### Partner TF *cis*-motif clusters are enriched in TF targets

We next sought to identify putative TFs partners that may work together with the 33 N-early response TFs to coordinate gene regulation in the dynamic N × Time response network. To do this, we looked for enrichment of *cis*-motifs for any TF in direct regulated targets of the 33 TFs. Given the large number of plant TF-binding *cis*-motifs from high-throughput methods such as DAP-seq^5^ and PBM^38,39^, and the fact that TFs from the same family often have similar *cis*-motifs, searching for each of the 1282 available *cis*-motifs is impractical. Therefore, we used the RSAT matrix-clustering tool^41^ on all of these known *cis*-motifs and identified 80 *cis*-motifs clusters (Supplementary Fig. 10). *Cis*-motifs from TFs belonging to the same family generally fell into the same *cis*-motif cluster, as seen previously for smaller sets of motifs^5,42^. For each *cis*-motif cluster, we obtained a consensus *cis*-motif (CCM) and corresponding position weight matrix (PWM)^41^ (Supplementary Data 8 and 9).

**Fig. 5:**
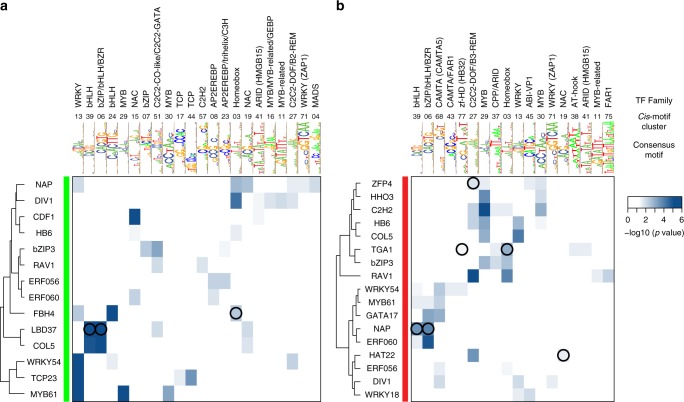
Direct regulated transcription factor (TF) targets are enriched for *cis*-motif clusters of partner TFs. Heatmap of enriched consensus *cis*-motifs for the 80 *cis*-motif clusters (columns) in the 500 bp promoter of the **a** induced and **b** repressed direct regulated targets of the 33 nitrogen (N)-early response TFs. The consensus *cis*-motif (CCM) logo, cluster number, and family representation for each CCM is shown above. Instances where there is a validated interaction between the TF under study and another TF within a family represented by the enriched CCM cluster are marked by a black circle. Of the 33 TFs validated in TARGET, only TFs with enrichment of any of the 80 CCMs in the 500 bp promoter are shown. *Cis*-binding motif clusters were determined using *cis*-motifs for *Arabidopsis* transcription factors collected from DNA affinity purification sequencing (DAP-seq)^5^, Cis-BP^38^, and protein binding microarrays (PBM)^39^. Blue shading represents *p* values calculated using Fisher’s exact test and false discovery rate (FDR) corrected. Source data of Fig. 5a, b are provided as a Source Data file

Using the PWM for each of the 80 *cis*-motif clusters, we looked for enrichment of each CCM in the 500 bp promoter (Fig. 5) and gene body (Supplementary Fig. 11) of the induced vs. repressed direct regulated targets of the 33 TFs. This analysis uncovered *cis*-motif enrichment in at least one of these regions for 30/33 TFs. Often, an enriched CCM represented a cluster for a TF family different from the TF tested in TARGET itself, pointing to the involvement of putative TF–TF interactions in gene regulation. This hypothesis is supported by validated protein–protein TF interactions^43–47^ between several of the 33 TFs assayed in TARGET and TF family members from the other *cis*-motif groups revealed by CCM enrichment analysis (Fig. 5 and Supplementary Fig. 11, black circles).

### Functional validation of a time-inferred N-response network

We next sought to expand our GRN of the N × Time response in roots beyond the direct regulated targets of the 33 N-early response TFs validated using TARGET (Fig. 2). To do this, we used our validated TF–target data to refine the precision of a GRN predicted from fine-scale time-series transcriptome data of N treatment in roots^29^ using dynamic factor graphs (DFG)^48^. DFG is a machine-learning method that can use time-series data to estimate the quantitative influence of TFs at time *t* on target genes at time *t* + 1^48^. This DFG approach has been used to learn network models that can predict gene expression states at future time points, even when few time points are tested^29,49^. In our application, the resulting DFG predictions provided an edge score, or measure of influence, for 145 TFs on every target gene in the root N × Time network, totaling 211,410 TF–target edges.

**Fig. 6:**
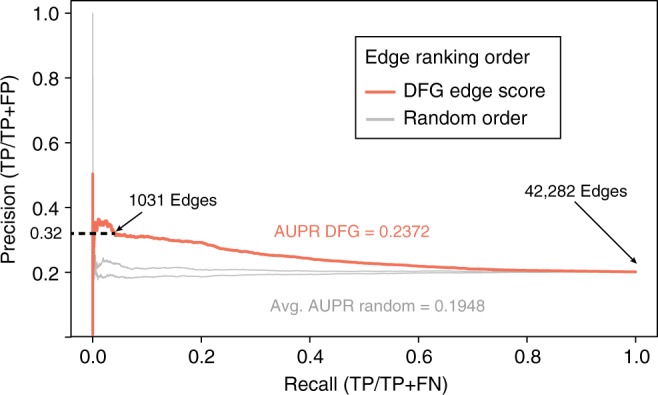
Validation of a time-inferred network using area under precision recall analysis. Genome-wide regulated targets of 29 transcription factors (TFs) captured in root cells using the TARGET (Transient Assay Reporting Genome-wide Effects of Transcription factors) system (Fig. 2, Supplementary Data 2) were used to calculate the precision and recall of the dynamic factor graph (DFG) inferred gene regulatory network (GRN) based on N × Time response genes in roots. Area under precision recall (AUPR) analysis demonstrates that the ranking of edges in the DFG time-inferred network (orange line) is significantly better (*p* value *<* 0.001, permutation test) than randomizing the order of edge rankings (*n* = 1000) (gray lines represent random networks with highest and lowest AUPR). From this plot a 0.32 precision cut-off was chosen as the highest value before the curve flattens. TP true positives, FP false positives, FN false negatives. Source data are provided as a Source Data file

To refine these time-based TF–target predictions, we used 71,836 validated TF–target edges for 29/33 TFs to calculate a precision threshold for the DFG predicted edges. This enabled us to set an edge score to prune the DFG network^1^ and retain only high-confidence TF–target edges involved in the root N response. Four of our 33 TFs—ZFP4, HSFB2A, TCP23, and HB6—were excluded from this analysis, as they did not meet the stringent threshold (FDR < 0.01) used to select the N × Time genes for DFG predictions^29^. The results of this precision/recall (PR) analysis showed that the area under precision recall (AUPR) for the TF–target predictions in the DFG predicted GRN (0.2372) was significantly greater than for 1000 random PR curves (mean = 0.1948) (Fig. 6 and Table 2). From the PR curve generated using the validated edges of 29 TFs, we chose a precision threshold cut-off of 0.32—the point at which the curve begins to flatten out—to filter the GRN for high-confidence TF–target edges. Our precision cut-off score of 0.32 (i.e., ~1/3rd of predicted edges are validated) is of comparable scale to the 0.50 precision achieved using an ensemble approach of multiple network inference methods in simpler microbial systems^1^. At this precision cut-off, the resulting pruned GRN was comprised of 6863 total high-confidence edges between 145 TFs and 311 targets in the root N × Time response network (Table 2, Supplementary Fig. 12 and Supplementary Data 10).

**Table 2:** Precision and recall pruning of a time-inferred network using TF–target validation

Validated network measures	Value
Area under precision recall (validated network)	0.2372
Area under precision recall (random network average)	0.1948
Validated AUPR *p* value (permutation test)	<0.001
Precision threshold for network pruning	0.32
Edge score threshold for pruned network	0.88242
Number of edges (pruned/total)	6836/211,410
Number of TFs (pruned/total)	145/145
Number of targets (pruned/total)	311/1458

*TF* transcription factor, *AUPR* area under precision recall

To evaluate the individual contribution of each TF to the edge pruning in the GRN, we calculated precision, recall, and *F*-score (harmonic mean of precision and recall^50^) for each of the 29 TFs within the pruned DFG network individually (Supplementary Table 3). While there was variation in all three metrics for each of the 29 TFs, the mean precision, recall, and *F*-score among all 29 TFs was 0.393, 0.149, and 0.17, respectively (Supplementary Table 3). These values are close to weighted values for the precision, recall, and *F*-score calculated from the TARGET edges for all 29 TFs combined (Supplementary Table 3). This result indicates that the collective PR measures are not biased towards a few TFs with many edges. Importantly, the 95% confidence intervals for precision (0.320–0.465), recall (0.064–0.204), and *F*-score (0.14–0.20) indicate that the TF–target edges predicted by DFG for the remaining 116 TFs in the N × Time network and their 311 targets are also likely to be true for ~1/3rd of the high-confidence TF–target edge predictions.

### Network Walking charts paths from direct to indirect targets

**Fig. 7:**
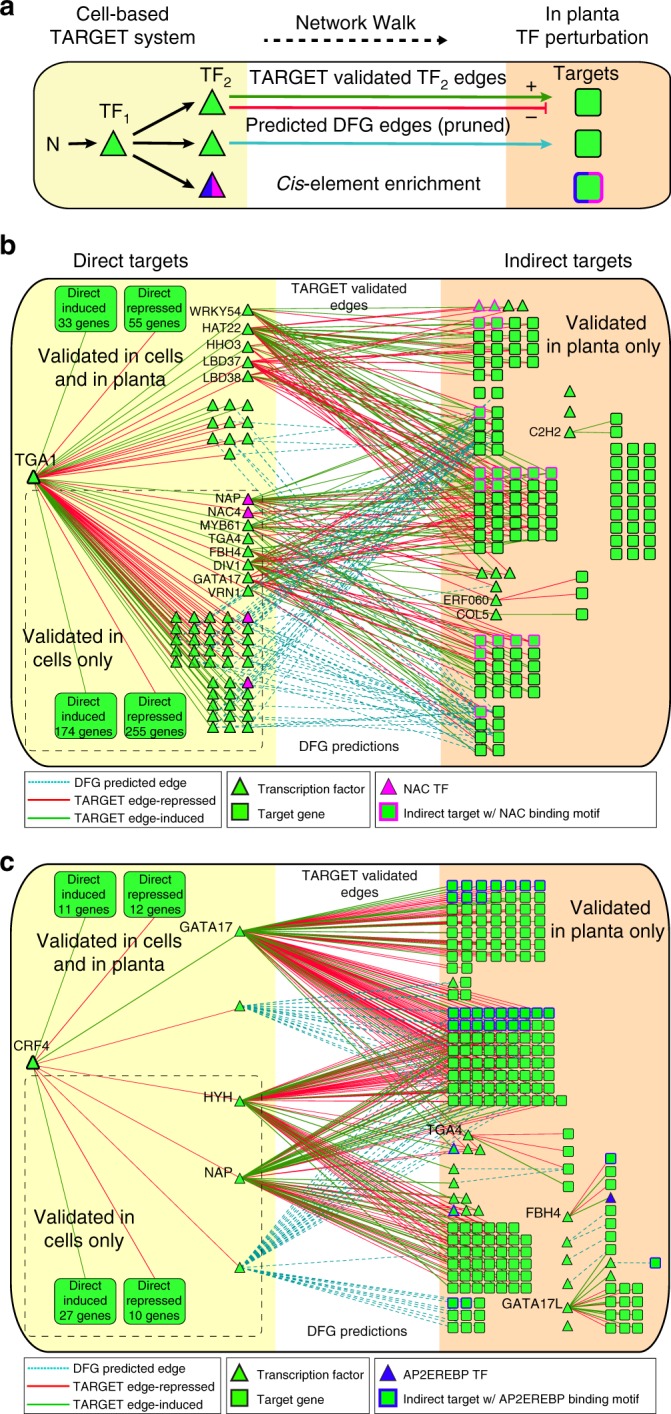
Network Walking charts a path from direct to indirect transcription factor (TF) targets. **a** A schematic representation of the Network Walking approach used to connect direct TF targets identified in cells to the indirect targets identified only in planta. Example of Network Walks from direct targets identified in cells (yellow shaded region), to indirect targets identified only in planta (orange shaded region) for **b** TGA1 and **c** CRF4. Edges connecting the indirect targets back to TGA1/CRF4 through their direct TF_2_ targets come from validated TARGET (Transient Assay Reporting Genome-wide Effects of Transcription factors) edges as well as from high-confidence edges from the pruned time-inferred dynamic factor graph (DFG) network. Enrichment of the consensus cis-motif for the 80 clusters (Supplementary Fig. 10 and Supplementary Data 8) in the 500 bp promoters and gene body of the indirect targets of TGA1 and CRF4 was assessed. The most significant cluster onsensus *cis*-motif (CCM) in indirect targets of TGA1 was for *cis*-motif cluster 15 (NAC family) in the gene body. For CRF4, the CCM for cluster 8 (AP2EREBP) was enriched in the gene body of CRF4 indirect targets. The network shown is limited to TFs and targets that respond to N × Time in Varala et al.^29^. For clarity, edges to target genes include only the top three validated edges based on fold change, and top ten predicted DFG edges based on edge score

Finally, we performed an analysis that integrates the validated TF–targets edges for 33 N-early response TFs from TARGET with the high-confidence edges for the 116 untested TFs in the pruned GRN and in planta TF perturbation data. In an approach called Network Walking (Fig. 7a), we used these combined datasets to chart a path for a TF_1_ from its direct regulated targets in root cells, to its indirect regulated targets in planta via intermediate TF_2_s. As proof of concept, we demonstrated how Network Walking revealed the network paths and mode of action for two important regulators of the N response in planta—TGA1^26–28^ and CRF4^29^ (Fig. 7b, c). The TF perturbation data used in this Network Walking analysis included direct targets that respond to the TF in cells (e.g., in TARGET) (Supplementary Data 2), and those that respond to TGA1 (this study, Supplementary Data 4) or CRF4 overexpression in planta (Varala et al.^29^, Supplementary Data 11).

In Network Walking, the first step is to use the direct regulated targets of TF_1_ (e.g., TGA1 or CRF4) identified using the TARGET assay to identify which DE genes from in planta perturbation are direct vs. indirect TF_1_ targets. In the TGA1 example, the TARGET assay showed that TGA1 directly regulated 580 root N × Time genes (Fig. 7b, yellow box), including 104 direct target genes that also respond to *TGA1* overexpression in planta. The second step is to connect a path from TF_1_—via a TF_2_—to the TF_1_ indirect targets which only respond in planta. To connect TGA1 to its indirect targets, we used validated TF_2_ direct target edges from TARGET assays (Fig. 2), as well as the high-confidence TF_2_target edges for 116 TFs from the pruned DFG network (Supplementary Fig. 12 and Supplementary Data 10). Using this approach, we could link 76% of indirect TGA1 targets in planta (101/133) back to TGA1 through 49/63 direct TF_2_ targets of TGA1. The set of 63 direct TF_2_ targets of TGA1 includes 13 TF_2_s whose direct regulated targets have been validated in TARGET (Fig. 2 and Supplementary Data 2), and 36 TF_2_s with high-confidence DFG predicted edges to indirect targets of TGA1. To further determine which of these intermediate TF_2_s are most important in relaying the N signal downstream of TGA1, we used the 80 *cis*-motif clusters (Supplementary Fig. 10 and Supplementary Data 8 and 9) to perform analysis of CCM enrichment in TGA1 indirect targets. This analysis showed that the most enriched CCM in the TGA1 indirect targets corresponds to cluster 15 (NAC family) which is enriched in the gene body (FDR = 7.9E-5, Fisher’s exact test) of TGA1 indirect targets.

We also performed a similar Network Walk for CRF4, and found that—by contrast to TGA1—relatively few N × Time genes are directly controlled by CRF4 (65 genes), yet the number of N × Time root genes that respond to CRF4 overexpression in planta (247 genes) is similar to the number responding to TGA1 overexpression in planta (208 genes) (Fig. 7b, c). In the CRF4 Network Walk, we identified direct connections of CRF4 to only 5 intermediate TF_2_s (GATA17, NAP, HYH, MYB34, bHLH112), which in turn accounted for the regulation of 87% of CRF4 indirect targets in planta. Thus, a Network Walk not only identifies the network path for each TF_1_ via its downstream TF_2_s, but can also help classify TF modes of action, as discussed below.

## Discussion

An ultimate goal of systems biology is to learn GRNs and infer TF–target models that can accurately predict future gene expression states under untested conditions. A key step to accomplish this is the experimental validation of edges between TF regulators and their target genes to use in refinement or as priors in network inference. Our study addresses the challenge of identifying functional in vivo targets of a TF genome-wide in a rapid and medium-throughput manner. We also demonstrate that the direct regulated targets of a TF identified using TARGET can enhance our understanding of TF-DNA binding data. For example, we found that in vitro high-throughput methods for identifying TF binding can overestimate (DAP-seq^5^) or underestimate (Y1H^28^) the number of functional TF–target interactions identified using the TARGET assay (Supplementary Fig. 9 and Supplementary Data 3). Similarly, in vivo ChIP assays are a poor predictor of TF-mediated gene regulation^8–10^. We demonstrate how direct regulated TF–target edges can be used to filter network predictions. Specifically, we used 71,836 validated targets for 29/33 N-early response TFs to prune a GRN predicted using DFG^48^ to obtain a refined N × Time GRN, where ~1/3rd of the edge predictions are likely true (Supplementary Fig. 12 and Supplementary Data 10). This approach enabled us extend our TARGET results beyond the 33 TFs to the remaining 116 N × Time TFs for which we do not yet have validated edges, and identify which TFs to target for further study based on their relative influence on the N × Time network.

To integrate our TARGET results with in planta data, we derived an approach called Network Walking (Fig. 7). The key feature of the Network Walking strategy is to connect the direct regulated TF_1_ edges identified in plant cells using TARGET to the indirect TF_1_ targets validated only in planta. This approach enabled us to identify the intermediate TF_2_s important for mediating the signal between the initial TF_1_ and downstream in planta indirect targets. The results can guide combinatorial experiments (e.g., TF stacking) and validation experiments on important TF_2_s that are identified in a systems biology cycle.

As proof of concept, we demonstrated the Network Walking approach for two TFs, TGA1 (Fig. 7b) and CRF4 (Fig. 7c), which are validated in planta regulators of the N response in *Arabidopsis*^26–29^. Our Network Walking approach showed that TGA1 directly regulates 40% (508/1458) of the N × Time genes in roots, including 63/145 N-responsive TF_2_s, amplifying the effect of TGA1 on the N response gene network. Moreover, our finding that CCM for the NAC family cluster is enriched in TGA1 indirect targets suggests that NAC TFs are particularly important TF_2_s for propagating the N signal downstream of TGA1.

The second example of Network Walking was for CRF4 (Fig. 7c), a TF which was recently shown to be involved in N signaling in shoots and roots in planta^29^. The TARGET data showed that direct regulated CRF4 targets are specifically enriched for the shoot N × Time-responsive genes (Fig. 2). Our Network Walk now resolves how CRF4 can have a significant influence on root N-responsive genes in planta (Supplementary Data 11), despite directly regulating only a small number of root N × Time genes itself (Fig. 2). This is because CRF4 directly regulates 5 TF_2_s which collectively have edges validated by TARGET and/or high-confidence predicted DFG edges to 50% of the root N × Time genes. Thus, the effect of CRF4 on roots in planta is mediated through these intermediate TF_2_s.

Overall, the 85,144 validated targets of 33 N-early response TFs revealed a connected GRN regulating 88% of the genes that respond to N treatment as a function of time in whole roots and shoots^29^, and the TFs collectively regulated a set of genes enriched in N-related processes (Fig. 3b, Supplementary Data 5). We also observed that the distribution of edges in the validated TF–target network (Fig. 3a) resembles a scale-free network^51^, a feature of biological networks that provides robustness^52^. Signal integration, an emergent property of biological systems^53^, could explain the large number of N-responsive TFs in shoots and roots (258 TFs), roughly 10% of all the predicted *Arabidopsis* TFs^29^. Indeed, many of the 33 TFs functionally validated in our study have defined roles in other pathways (Supplementary Data 12), linking the N response to other biological processes, including hormone signaling^54^ and biotic stress^55^. These types of complex combinatorial interactions between TFs, which integrate or fine-tune a response to signal inputs^56^, have been described in many organisms^57–59^.

Because TARGET is a cell-based TF perturbation system, direct regulated targets identified in vivo can provide biological context to TF-binding data. Somewhat surprisingly, our analysis revealed that all 33 of the TFs we assayed acted as both an inducer and repressor of direct regulated targets, and that *cis*-binding motifs for a TF are often specific to a particular direction of regulation (Fig. 4). Particularly interesting are group III TFs, where the pattern of enrichment of the known *cis*-motifs suggests that direct TF binding in the promoter leads to induction while direct binding to the gene body leads to repression. We also found that the direct regulated targets of TFs that could not be explained by TF binding to the known *cis*-motif for that TF (based on in vitro TF-DNA binding data) may involve partner TFs which are not present in in vitro binding assays. Indeed, our *cis*-motif cluster analysis (Fig. 5 and Supplementary Fig. 11) addressed the duality of TF function (e.g., inducer and repressor) observed in the regulatory action of each of the 33 TFs. We noted several instances where the direct TF targets were enriched in CCMs for TF families other than the TF under investigation (Fig. 5 and Supplementary Fig. 11). This finding could explain how the same TF could mediate repression of direct targets (via a partner TF-binding site) vs. induction when the TF binds directly to DNA. As one example, the *cis*-binding motif for NAP is enriched only in its induced targets (Fig. 4), while the repressed NAP targets are instead enriched in bHLH sites (clusters 6 and 36) in the 500 bp promoter. Moreover, the experimentally validated interaction between NAP and a bHLH family TF—bHLH96^43^—could explain how NAP is able to repress its direct target genes via its interaction with bHLH96. This model is also supported by the finding that protein–protein interactions between TFs have been shown to alter the effect of a TF on target gene expression^60–62^.

The development of tools for validating the TF–target edges within inferred networks is crucial to obtaining high-quality predictive GRNs. In this study, we demonstrate that the TARGET system for cell-based TF perturbation complements existing TF–target binding approaches and in planta perturbation by providing direct TF–target edges based on TF-mediated gene regulation in cells isolated from the tissue of interest. Importantly, this system does not require the creation of stable transgenics and scales easily. We have also introduced an approach, Network Walking, which connects the early and direct regulated TF targets identified in cells using TARGET to downstream responses observed only in planta. While our proof-of-concept studies focused on rapid N signaling in *Arabidopsis*, both of these approaches are generally applicable to study of GRNs involved in transducing signals in any eukaryotic system in agriculture, biology, or medicine.

## Methods

### A scaled-up TARGET assay for TF perturbation in cells

To make the TF-plasmid constructs, the 33 N-early response TFs were TOPO cloned into pENTR (Invitrogen) from complementary DNA or isolated from the *Arabidopsis* TF collection^63^. TFs were then transferred to the pBeaconRFP_GR plasmid^18^ or a GFP version of the same plasmid (pBeaconGFP_GR) by Gateway (Invitrogen) cloning.

For protoplasting and transfections, *Arabidopsis* Col-0 plants were grown in 1% w/v sucrose, 0.5 g per L MES, 1× MS basal salts (-CN), 1 mM KNO_3_, 2% agar, pH 5.7 for 10 days prior to the TARGET experiment. Light conditions were 120 μmol m^−2^ s^−1^ at constant temperature at 22 °C, 16 h light, 8 h dark (long day). Roots of 10-day-old seedling were harvested and the cell wall removed using cellulase and macerozyme (Yakult, Japan) for 3 h. Cells were filtered sequentially through 70 µm and 40 µm cell strainers (BD Falcon, USA) and pelleted at 500 × *g*. Filtered cells were washed with 15 mL MMg solution (400 mM mannitol, 10 mM MgCl_2_, 4 mM MES pH 5.7), resuspended to approximately 2–3 × 10^6^ cells per mL. For each transfection, in a 50 mL conical tube, 1 mL of cell suspension was mixed with 120 μg of plasmid DNA, 1 mL of PEG solution (40% polyethylene glycol 4000 (Millipore Sigma, USA), 400 mM mannitol, and 50 mM CaCl_2_) and vortexed gently for 5 s. After mixing, 50 mL of W5 buffer (154 mM NaCl, 125 mM CaCl_2_, 5 mM KCl, 5 mM MES, 5 mM glucose, pH 5.7) was slowly added to the tube. Cells were pelleted at 1200 × *g*, and washed 3 times with W5 buffer. For each TF and the EV construct, 4–6 million cells were transfected and after washing, a single TF in the RFP vector and a single TF in the GFP vector were aliquoted into 3 replicate wells of a 24-well plate. After overnight incubation, each pool of transfected root protoplasts was treated with the N dose present in standard MS media^64^ (20 mM KNO_3_ + 20 mM NH_4_ NO_3_) for 2 h. Next, 35 µM CHX was added 20 min before a 10 µM DEX treatment. Transfected cells were sorted by FACS into GFP- and RFP-expressing populations 3 h after DEX treatment.

For transcriptome analysis, cells expressing the candidate TF or EV were collected in triplicate and RNA-Seq libraries were prepared from their mRNA using the NEBNext® Ultra™ RNA Library Prep Kit for Illumina®. The RNA-Seq libraries were pooled (up to 27 libraries per run) and sequenced on the Illumina NextSeq 500 platform. The RNA-Seq reads were aligned to the TAIR10 genome assembly using TopHat2^65^ and gene expression estimated using the GenomicFeatures/GenomicAlignments packages^66^. The gene counts for every sample were combined and DE genes in the TF transfected samples vs the EV samples were identified using the DESeq2 package^67^ with a TF+Batch model and an FDR adjusted *p* value < 0.05. We filtered out genes that respond more than 5-fold to CHX treatment in transfected protoplasts from the lists of TF targets (Supplementary Data 1 and Supplementary Methods). Genes that are expressed in any of the protoplast experiments (excluding the CHX-responsive genes in Supplementary Data 1) were used as the background for subsequent enrichment analyses.

### Calculating nitrogen specificity and influence of TF targets

The specificity of each TF to target genes in the N × Time-responsive dataset of Varala et al.^29^ was calculated by dividing the N × Time-responsive targets of a TF by the total number of targets regulated by that TF. The influence of a TF on the N × Time-responsive genes is the number of N × Time-responsive genes targeted by a TF divided by the total number of N × Time-responsive targets. The N-specificity index *p* value^29^ was calculated using the one proportion *z*-test to compare the proportion of targets for a TF in the genome to the proportion of targets for that TF in the root N × Time-responsive genes^29^, under the null hypothesis that they are equal.

### GO enrichment in TF–target networks

The web application agriGO v2.0^68^ was used to identify GO terms enriched in the cumulative direct regulated targets of the 33 TFs. To calculate the GO term enrichment in the sets of targets regulated by increasing number of TFs (Fig. 3b), let *G* be a list of genes associated with a GO term and *T*_*k*_ be a list of targets that are targeted by at least *k* TFs. We can calculate the enrichment of *G* (*E*_*g*_) in *T*_*k*_ by simply comparing the frequency of *G* in *T*_*k*_ (i.e., the fraction of genes in *T*_*k*_ that intersect *G*), represented as *F*_*k*_, to the frequency of *G* in the background (all 20,662 genes expressed in protoplast experiments), represented as *F*_*b*_.

1 $$E_g = \frac{{F_k}}{{F_b}}.$$

To test for the significance of this enrichment, we used the figure of merit Focus. For each gene list (*G*) we calculate Focus (*F*_*g*_) by simply adding the *E*_*g*_ for all *k* up to *n* TFs:

2 $$F_g = \mathop {\sum }\limits_{k = 1}^n \frac{{F_k}}{{F_b}}.$$

To test if the Focus of the validated network is significant for a given GO term, a permutation test was used to determine an empirical *p* value by comparing the Focus of the validated network to the Focus of 1000 iterations of a randomized network, generated by shuffling the edges within the experimentally validated TF–target network.

### *Cis*-motif enrichment and clustering

Enrichment of the *cis*-binding for motifs in TF target genes was calculated using the AME tool within the MEME package^69^. The background used corresponded to the same gene region for all genes expressed in any of the cell-based TARGET experiments and the frequency of bases to the base frequency within the background.

For *cis*-motif clustering, *cis*-binding motifs for *Arabidopsis* transcription factors were collected from DAP-seq^5^, Cis-BP^38^, PBM of Franco-Zorrilla et al.^39^ and ChIP-seq from Song et al.^37^. PWMs were converted to the MEME motif format^70^ and the RSAT matrix-clustering tool^41^ was used with the following parameters: hclust_method = average, calc = sum, metric_build_tree = Ncor, lth w 5 lth cor = 0.6, lth Ncor=0.45, quick=true. To search for the enrichment of each *cis*-motif in the TF targets, the consensus PWM for each of the 80 *cis*-motif clusters was converted to the MEME format and the FIMO tool within the MEME package^69^ was used to identify every occurrence of each of the 80 consensus *cis*-motifs in the 500 bp promoters and gene body of all 20,662 protoplast expressed genes at a *p* value < 0.0001. Overlapping *cis*-motifs were removed, retaining only the *cis*-motif with the lowest *p* value. For each set of TF targets, enrichment of a *cis*-motif in the target set relative to their occurrence in all annotated genes was calculated using Fisher’s exact test. The resulting *p* values were FDR corrected. Heatmaps and hierarchical clustering were generated with Euclidean distance and the ward.D agglomeration method using the gplots heatmap.2 function in R.

### Identification of direct and indirect TGA1 targets in planta

The in planta *TGA1* overexpression construct was made by Gibson assembly (NEB) with a three‐part construct. The CaMV‐35s promoter was fused to the *TGA1* CDS using in the pGreen vector backbone. Primers used in the assembly are in Supplementary Table 4.

*Arabidopsis* Col-0 plants with the *35S:TGA1* transgene were generated using Agrobacterium-mediated floral-dip method. Approximately 100 seeds were sown in Phytatrays (Sigma-Aldrich) in liquid media that was identical to what was used in the TARGET assay: 1% w/v sucrose, 0.5 g per L MES, 1× MS basal salts (−CN), 1 mM KNO_3_, pH 5.7. Light conditions were 120 μmol m^−2^ s^−1^ at constant temperature at 22 °C, 16 h light, 8 h dark (long day).

When *35S:TGA1* seedlings were 13 days old, they were transferred to N-starvation media (1% w/v sucrose, 0.5 g per L MES, 1× MS basal salts (−CN), pH 5.7). After 24 h, at 2 h after subjective dawn, seedlings were transferred to Phytatrays containing identical media with the addition of the N dose in standard MS media^64^ 20 mM KNO_3_+20 mM NH_4_NO_3_ or 20 mM KCl control. Plants were incubated within treatment media for 2 h after which root tissue was immediately harvested and flash frozen in liquid nitrogen.

RNA was extracted from root tissue using the QIAGEN RNeasy kit (Qiagen). mRNA was purified with oligo-dT beads (Invitrogen), and RNA-seq libraries made using the NEBNext Ultra Library Prep Kit (NEB). Libraries were sequenced the Illumina HiSeq 2500 v4 platform using 1 × 50 or 1 × 75 single end chemistry. RNA-seq reads were aligned as described for the protoplast samples and DE genes were identified using DESeq2^67^.

### Time-based network inference and AUPR validation

The time-based DFG network inference^48^ predicted GRN was generated using the N-treatment time-series data as described in Supplementary Methods. We used a pruning approach to filter this network for high-confidence edges^1^. The validated TF–target edges from TARGET were used to perform an AUPR analysis and identify a precision threshold of 0.32 (Fig. 6 and Table 2). This TF–target edge cut-off was chosen to minimize false positives, while recovering as many true positives as possible. The resulting pruned DFG inferred network was visualized (Supplementary Fig. 12) using Cytoscape^71^. Precision, recall, and *F*-score were calculated for the edges in the pruned network to generate Supplementary Data 10.

3 $${\mathrm{Precision = True}}\;{\mathrm{Positives/}}\left( {{\mathrm{True}}\;{\mathrm{Positives + False}}\;{\mathrm{Positives}}} \right).$$

4 $${\mathrm{Recall = True}}\;{\mathrm{Positives/}}\left( {{\mathrm{True}}\;{\mathrm{Positives + False}}\;{\mathrm{Negatives}}} \right).$$

5 $${\mathrm{F-score = }}\left( {{\mathrm{2}} \ast {\mathrm{Precision}} \ast {\mathrm{Recall}}} \right){\mathrm{/}}\left( {{\mathrm{Precision + Recall}}} \right).$$

### Reporting summary

Further information on experimental design is available in the Nature Research Reporting Summary linked to this article.

## Supplementary information

Supplementary Information

Reporting Summary

Description of Additional Supplementary Files

Supplementary Data 1

Supplementary Data 2

Supplementary Data 3

Supplementary Data 4

Supplementary Data 5

Supplementary Data 6

Supplementary Data 7

Supplementary Data 8

Supplementary Data 9

Supplementary Data 10

Supplementary Data 11

Supplementary Data 12

Source Data

## Data Availability

All raw sequencing data from this project have been deposited in the Gene Expression Omnibus (GEO) database accession GSE117857 and GSE128209. Data supporting the findings of this work are available within the paper and its Supplementary Information files. A reporting summary for this Article is available as a Supplementary Information file. The datasets generated and analyzed during the current study are available from the corresponding author on reasonable request. The source data underlying Figs. 3a, 3b, 5a, 5b, and 6, as well as Supplementary Figs. 2, 4b-d, 5, 6, 8, 9, 11a, and 11b are provided as a Source Data file.

## Code availability

The previously published dynamic factor graphs (DFG) network inference machine-learning code is available at [https://github.com/piotrmirowski/DFG4GRN]^49^. Python scripts used for calculation of motif enrichment and randomization the networks for calculating *p* values are available from the corresponding author upon reasonable request.
