# Supplementary material for: Network Walking charts transcriptional dynamics of nitrogen signaling by integrating validated and predicted genome-wide interactions
Source: Nat Commun. 2019 Apr 5;10:1569. doi: 10.1038/s41467-019-09522-1 (PMC6451032; doi:10.1038/s41467-019-09522-1)
Supplement: Supplementary file 1 — Supplementary Information [file 41467_2019_9522_MOESM1_ESM.pdf]

Network Walking charts transcriptional dynamics of nitrogen signaling by integrating validated and predicted genome-wide interactions

Brooks *et al.*

Supplementary Methods

Effects of treatment conditions in root protoplast

To test the effects of cycloheximide (CHX) on gene expression in isolated root cells transfected with an expression construct, we performed the *TARGET* assay (as described in the main text and Methods) with the following changes. A total of 8-12 million cells were transfected separately with either the GR-only empty vector (EV) or GR-TGA4, in pBeaconRFP_GR[1]. Cells transfected with each construct were split into 6 replicate wells of a 24 well plate after being washed three times. Following overnight incubation, transfected root protoplasts were treated as described in the main text (+N/+Dex) except that 20 min before DEX treatment half of the samples (3 replicates) for each construct were treated with 35uM CHX in DMSO (+CHX) and half with DMSO only (-CHX). Transfected cells were sorted by FACS for RFP expressing cells 3 hours after DEX-induced nuclear import.

The effect of nitrogen (N) pre-treatments on identification of TF-target genes was tested in the TARGET assay (as above) with the follow changes. Following transfection of 12-16 million cells with the same constructs, samples were split into 9 replicate wells in a 24 well plate. After overnight incubation and 2 hours before DEX treatment, root protoplasts transfected with each construct were treated with either 20mM NH_4NO_3 + 20mM NH_4KNO_3, 5mM KNO_3 + 15mM KCl, or 20mM KCl (3 replicates each treatment). All samples were treated with 35uM CHX for 20 min before DEX treatment.

For the above samples, libraries were generated, sequenced and processed as described in the main text. Genes that had a 5 fold-change difference in expression level in the +CHX condition compared the -CHX condition (Table S1) were excluded from subsequent analyses.

Nitrogen response in protoplasts compared to whole roots

To compare the N-response in the isolated root protoplasts transfected with an expression construct compared to whole roots, we grew and transfected protoplasts with the EV construct, as described in the main text. N-treatment of the protoplast samples was either 20 mM KNO3 + 20 mM NH4NO3 or 20 mM KCl. No CHX or DEX treatments were performed. In parallel, we grew Arabidopsis plants in Phytatrays (Sigma) in liquid media of the same composition (1% w/v sucrose, 0.5 g/L MES, 1X MS basal

salts (-CN), 1 mM KNO_3, pH 5.7). After 11 days, and at the same time, transfected root protoplasts were being treated with N, we transferred the Arabidopsis plants to fresh Phytatrays containing either the basal MS media (1% w/v sucrose, 0.5 g/L MES, 1X MS basal salts (-CN), pH 5.7) with N-supply (20 mM KNO_3 + 20 mM NH_4NO_3) or with 20 mM KCl. Five hours after N-treatment, when the protoplasts were being sorted, roots were harvested and flash frozen in liquid nitrogen. Four independent Phytatray and protoplasts samples were collected for each treatment. Libraries were generated, sequenced and processed as described in the main text.

Construction of a nitrogen-response inferred network

We used a machine learning approach called Dynamic Factor Graphs (DFG)[15], which we have previously validated[6,16,17], to derive the TF-target interactions in response to N-treatment in Arabidopsis roots. DFG infers interactions between 145 TFs and 1458 genes that responded to N in the root time-course[6]. DFG learns an *f* function to explain the target gene expression at each time-point, based on the expression of the TFs at previous time-points. This identifies the TFs influencing target gene expression. We use the time-series transcriptome data[6] to learn hyper-parameters of the DFG model using a leave-out time-point. Hyper-parameter optimization is the process of choosing a set of hyper-parameters for a good generalization of a learning algorithm[18]. DFG is trained on the first 9 time-points of the 10-time-point dataset, and tunes the hyper-parameters to minimize error on the remaining final time-point. The final matrix using all 10 time points estimates the influence of each TF on every N-responsive gene. This matrix is used to construct an inferred network where the coefficient of TF influence on each target gene is assigned as the edge score between that TF and target. This unpruned network generated by DFG contains an edge between every TF and every target gene (211,410 edges).

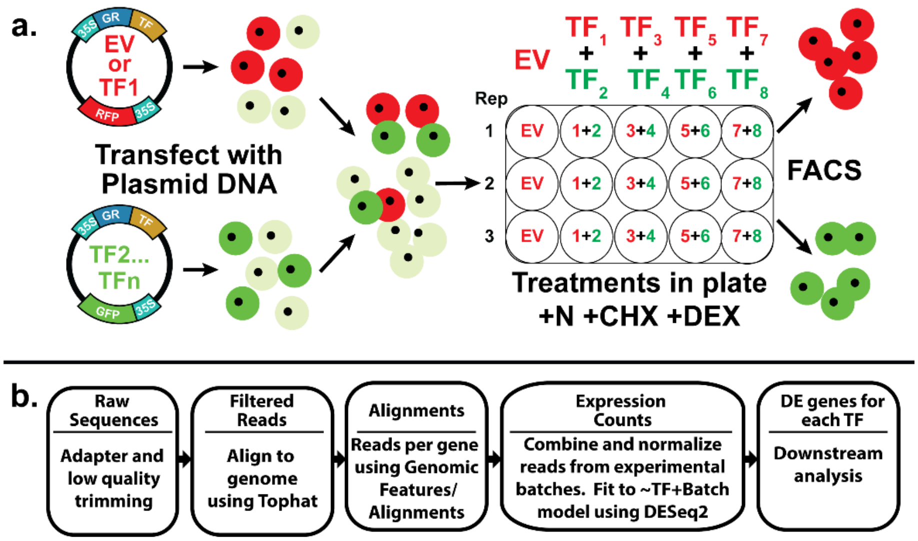

Supplementary Figure 1. Innovations in the cell-based TARGET system for TF perturbation to increase throughput for identifying direct regulated targets of N-early response TFs.

(a) Enhancement of throughput of TF perturbations performed in the cell-based TARGET assay[1] was done to enable concurrent processing of multiple TFs for validation screening up to 24 TF assays/ day (e.g. 8 TFs x three replicates). Use of separate vectors containing GFP (green) and RFP (red) reporters allows pooling of protoplasts transfected with the individual vectors into sample wells and reduces the time need for FACS. Transfection of cells with an Empty Vector (EV) enables identification of TF-regulated genes across multiple TF samples. FACS - Fluorescence Activated Cell Sorting, N - nitrogen, CHX - cycloheximide, DEX - dexamethasone. (b) General workflow for processing the RNA-seq results for the TARGET experiments with the input files at each step on top. After trimming adapter and low quality reads and bases, TopHat2[2] was used to align reads to the Arabidopsis TAIR10 genome. Gene counts for the aligned reads were estimated using the GenomicFeatures and GenomicAlignments[3] packages. Expression counts from samples were combined and analyzed using DESeq2[4]

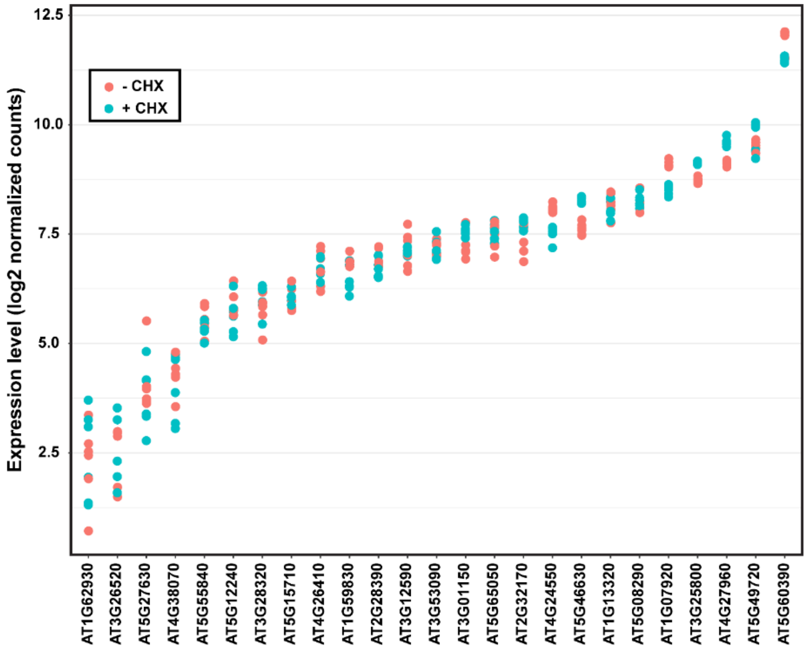

Supplementary Figure 2. The expression of 25 superior housekeeping genes monitors the global effects of cycloheximide treatment on RNA metabolism of root cells.

We tested the effect that cycloheximide (CHX) had on 25 superior housekeeping genes from Czechowski *et al.*[5], by performing parallel TARGET assays with and without the CHX treatment (6 treatment replicates). We found that while CHX does have an effect on some housekeeping genes (e.g. AT5G60390), the effect of CHX did not drastically change the expression level of the gene relative to other genes. These results suggest there is no global effect on RNA metabolism due to CHX treatment. Finally, the CHX treatment also had a consistent effect on each of the genes in each of the replicates, indicating that comparison of TARGET samples (EV and TFs), which have all been treated with the same amount of CHX, should not result in any biases. Red points – minus CHX samples, Blue points – plus CHX samples. Source data are provided as a Source Data file.

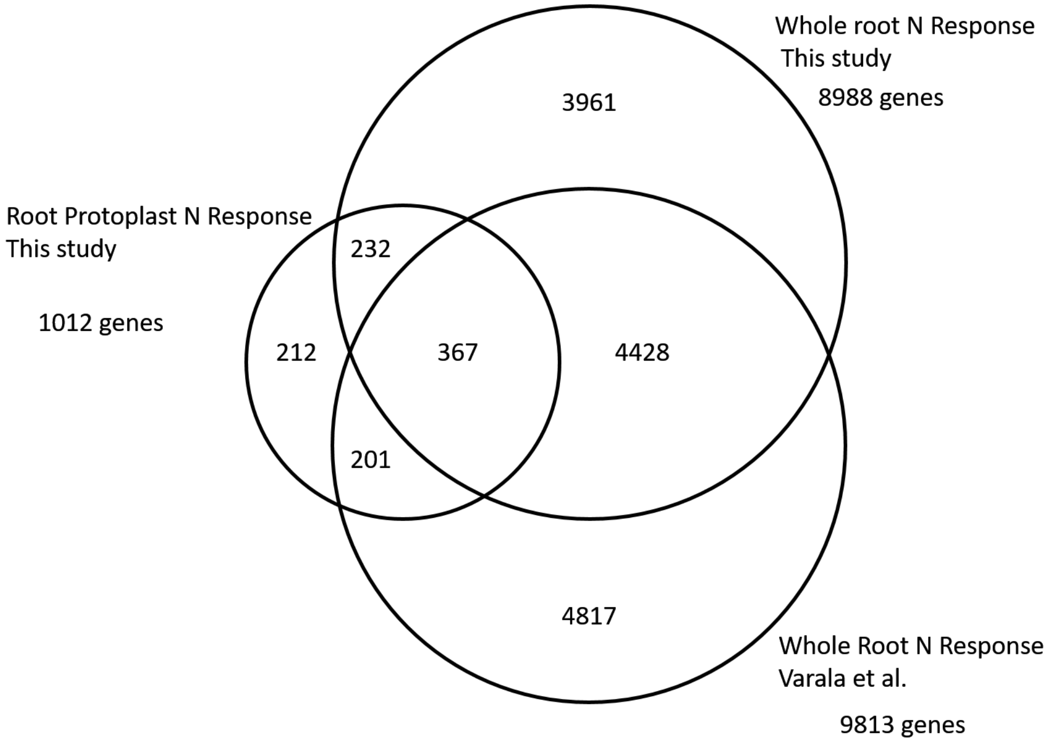

Supplementary Figure 3. The nitrogen responsive genes in isolated root cells overlaps significantly with N-response in whole roots.

Venn diagram analysis of the genes responding to N treatment in either isolated root cells that have been transfected with the empty vector construct or whole roots, either in this study (see Supplementary Methods) or in Varala *et al.* (2 hour treatment)[6]. This analysis shows that 80% of genes that respond to N-treatment in transfected root protoplasts respond in the whole root data sets. While a larger number of genes respond to N in intact roots, the overlap between the N-response genes in root protoplast and either whole root experiment (e.g. 56-59% of root protoplast N responsive genes) is similar to the overlap between the two whole root datasets (49-53%).

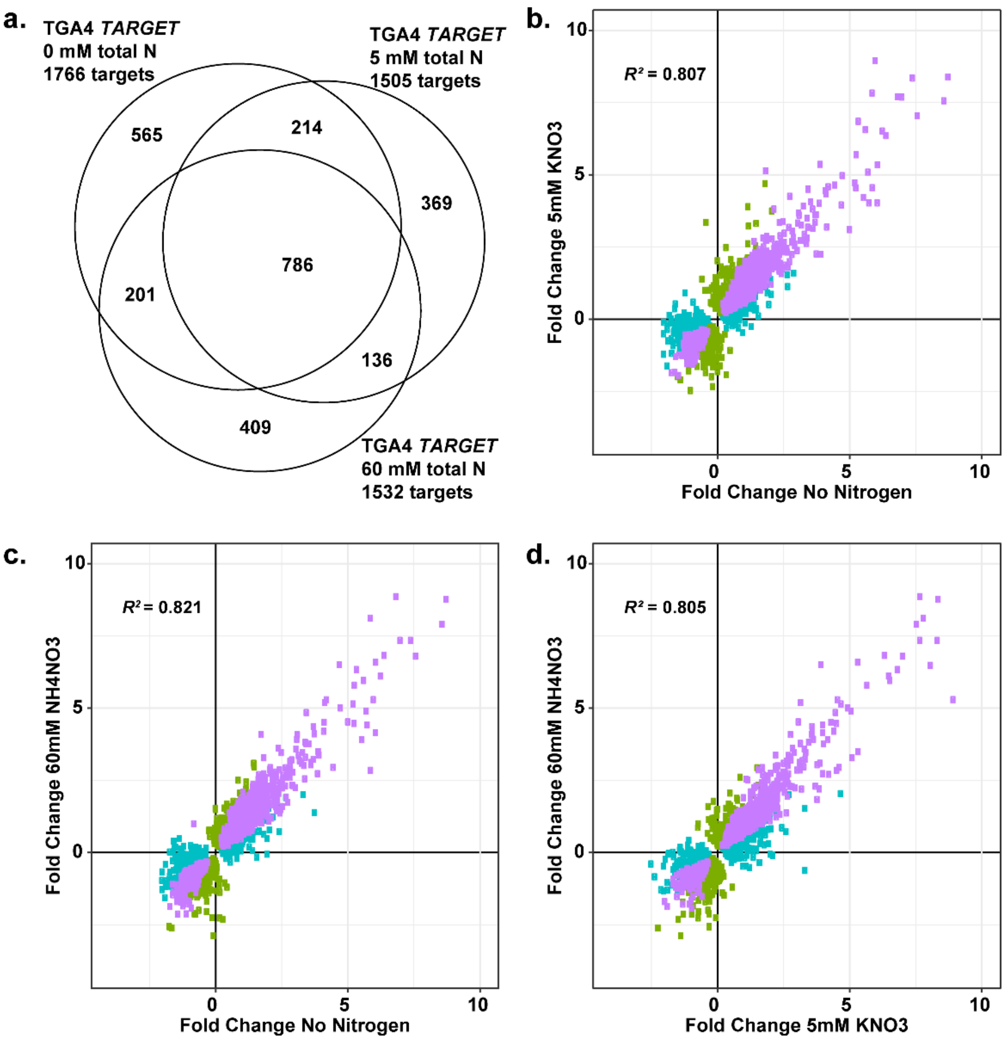

Supplementary Figure 4. The majority of TGA4 targets identified in root cells exposed to different nitrogen pre-treatments are conserved.

Parallel TARGET assays were performed on TGA4 where the total N dose (0mM, 5mM and 60mM total N) and source (no N, nitrate only, ammonia and nitrate) differed between experiments. (a) The total number of target genes identified in each case was similar and a majority of these were shared between experiments. (B-D) Scatter plots showing the fold change of target genes between treatments reveal that the effect of TF overexpression on most target genes is the same between different N pre-treatments, but the amplitude of the response for a fraction of the genes depends on N dose/source. Blue points and green points are differentially expressed only for N pre-treatment shown on X-axis or Y-axis respectively, while purple points are differentially expressed in both N pre-treatments. The 60mM N dose is the same as standard MS growth media[7] (20mM NH_4NO_3 + 20mM KNO_3). Source data Supplementary Figure 4b-d are provided as a Source Data file.

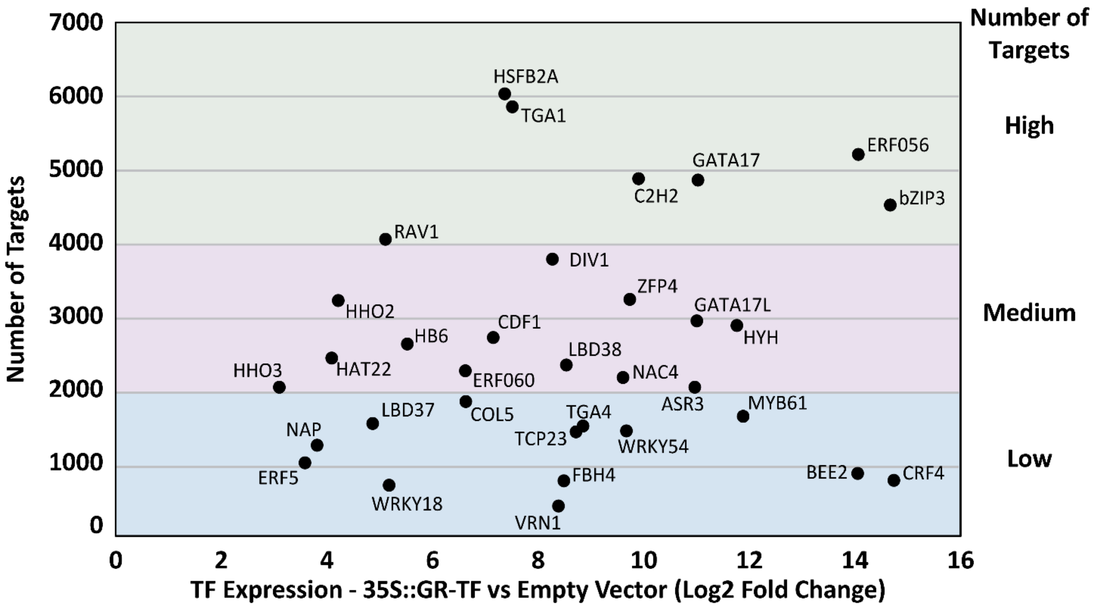

Supplementary Figure 5. The relative level of overexpression of a TF is not proportional to the number of target genes identified

To ascertain if high levels of overexpression of a TF in the TARGET system leads to more off-target genes responding to the TF, we plotted the level of TF expression relative to the empty vector control against the number of genes identified as differentially expressed. There is no correlation between expression level of the TF and number of target genes. Indeed, we see that two of the most highly over-expressed TFs, CRF4 and BEE2, have the fewest number of targets. Source data are provided as a Source Data file.

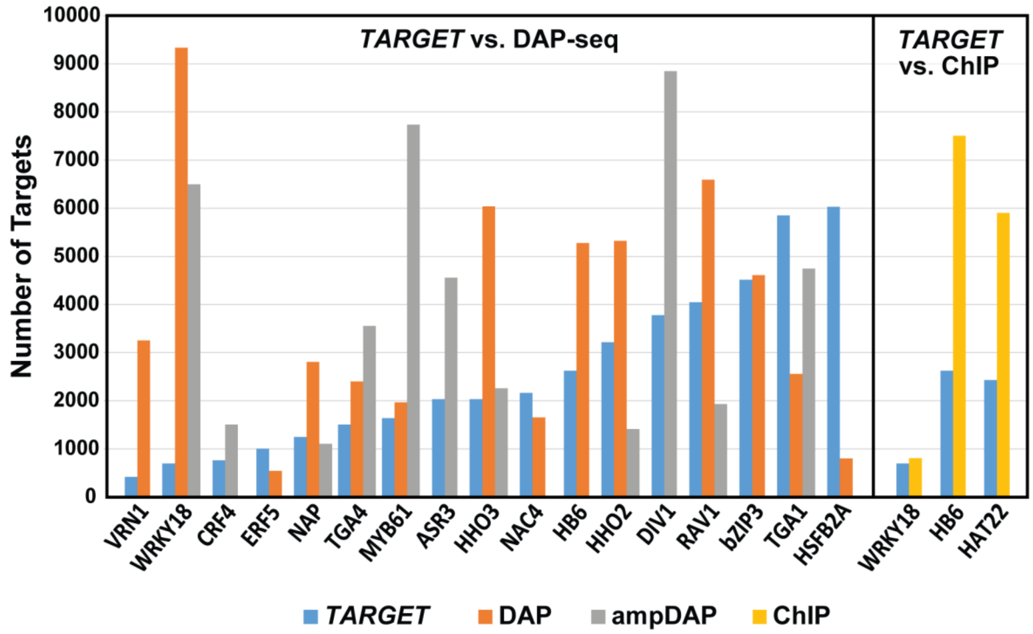

Supplementary Figure 6. The TARGET assay most often identified fewer direct regulated targets compared to TF-targets identified by *in vitro* or *in vivo* binding assays.

Barplots of the number of regulated target genes identified for each TF from TARGET, compared to the number of targets found to be bound using DAP-seq[8] (*in vitro)* and ChIP[9,10] (*in vivo).* The number of regulated TF targets identified in TARGET (blue bars) is typically lower than those detected by *in vitro* binding (DAP – red bars, ampDAP – grey bars) with a few exceptions (ERF5, HSFB2A, NAC4, and TGA1). The number of TF-targets identified by TARGET (blue bars) are lower than those detected by *in vivo* ChIP (yellow bars) experiments in all three examples. The overlap between direct regulated TF-targets identified in TARGET and DAP-seq bound targets is significant for 13/17 TFs (Supplementary Data 3) and 3/3 TFs for ChIP-seq experiments (Table 1). Source data are provided as a Source Data file.

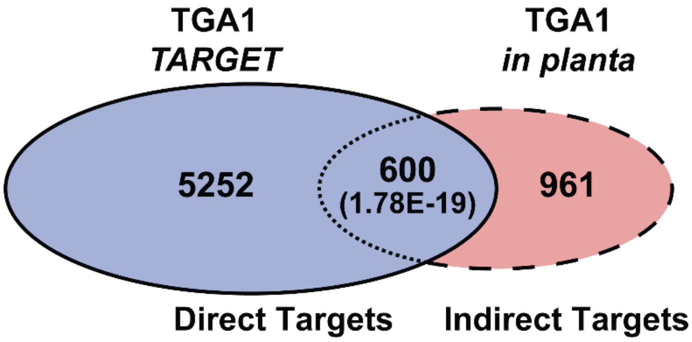

Supplementary Figure 7. Validation of the *in planta* relevance of direct TF-regulated targets identified in cells using the TARGET assay

The targets of TGA1 identified in isolated root cells using the TARGET assay (Supplementary Data 2) overlap significantly with the targets that respond to *TGA1* overexpression in whole roots (Supplementary Data 4). The number in each area represents the number of TGA1 targets identified, and the number in parenthesis is the p-value (Fisher's exact test) of the overlap between the TGA1 targets identified in root cells using TARGET and those identified by TF overexpression *in planta*. We can also identify candidate direct targets (blue shading) as those that respond to the TF perturbation in isolated root cells (in the presence of CHX), and genes which only respond to *TGA1* overexpression *in planta*, which are more likely to be indirect targets (red shading).

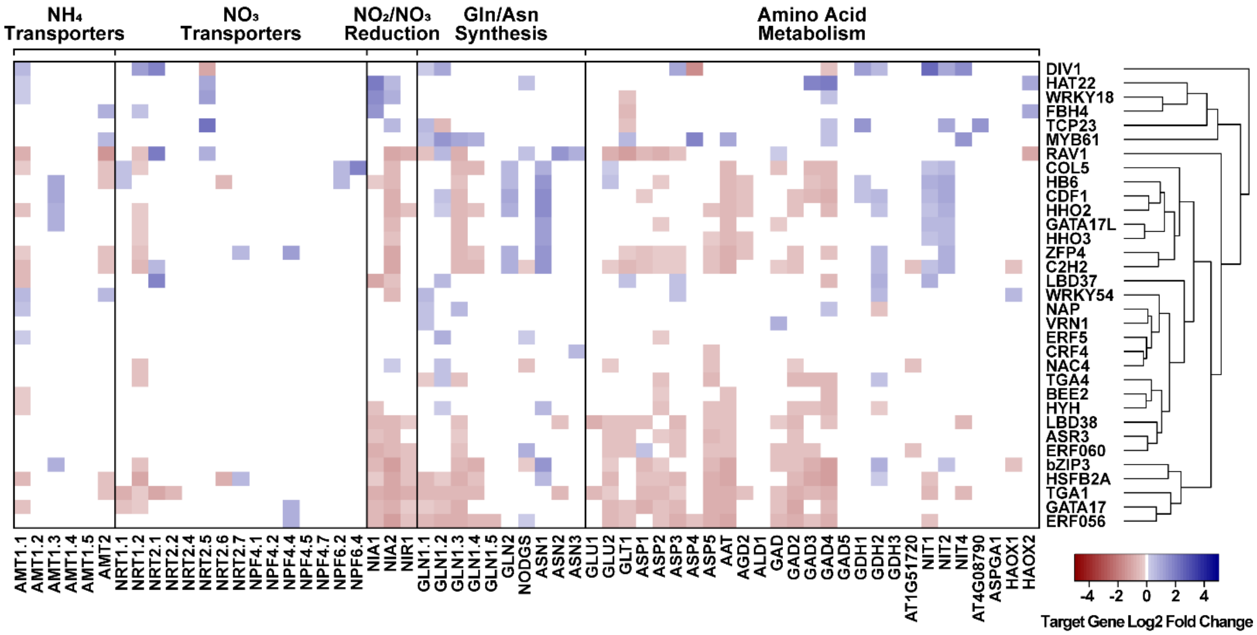

Supplementary Figure 8. Combinatorial influence of the N-early response TFs on N-uptake and N-assimilation pathway

Heatmap displaying the influence of each of the 33 N-early response TFs on the genes involved in N-uptake (ammonia and nitrate transporters), nitrate reduction to ammonia, Glutamine (Gln) and Asparagine (Asn) synthesis, and amino acid metabolism. The dendogram analysis clusters TFs that regulate shared genes in the pathway. Red and blue shading indicate repression and induction, respectively, relative to the EV control. Source data are provided as a Source Data file.

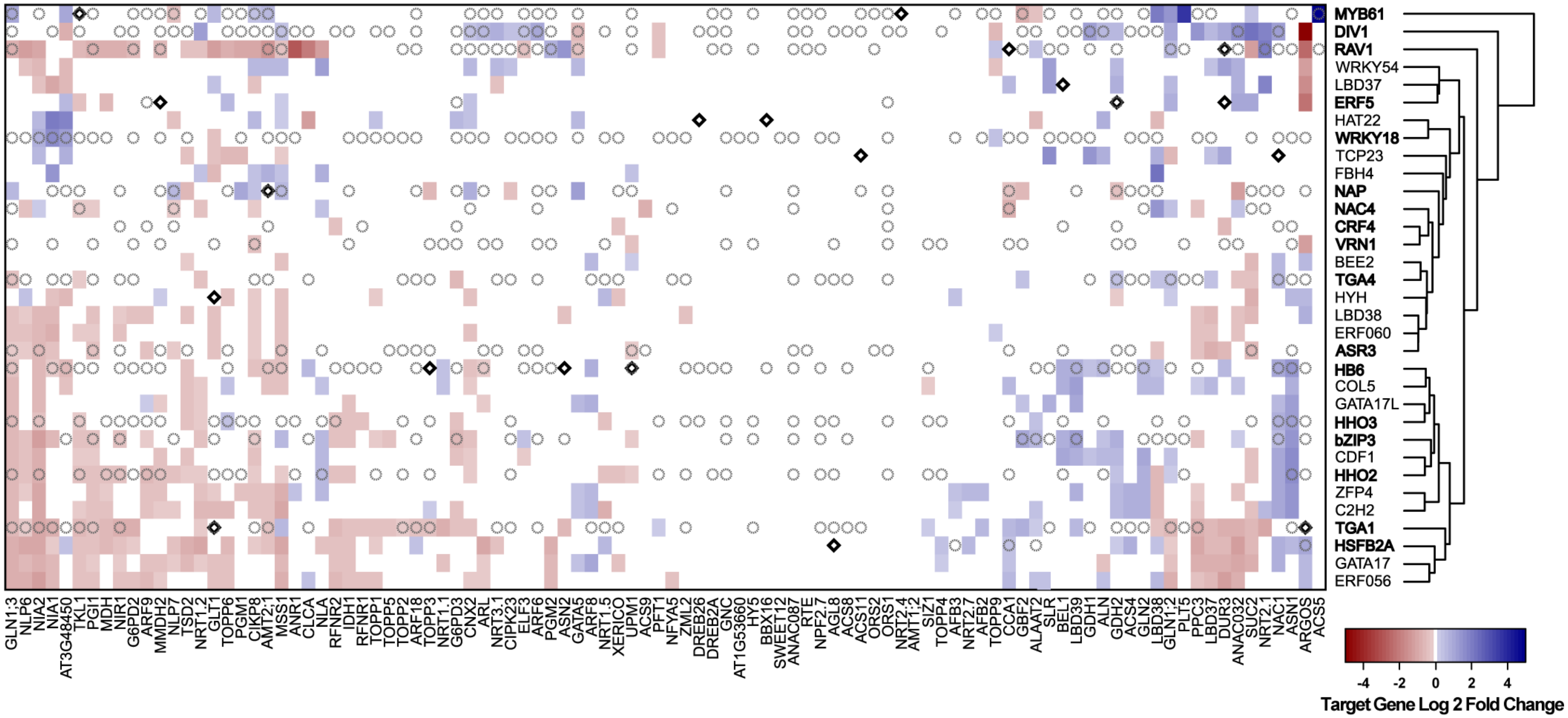

Supplementary Figure 9. Direct regulated edges to the N metabolism network identified *in vivo* by TARGET complement TF-binding assays Heatmap displaying the influence of each of the 33 N-early response TFs on the 98 genes in the N-metabolism network screened by Gaudinier *et al*. in a recent high-throughput yeast-one-hybrid (Y1H) study[11]. The TARGET assay identifies 425 direct regulated targets of these 33 TFs for this set of genes in Arabidopsis root cells, compared to the 20 edges identified in the Y1H study (open diamonds). We also looked at the number of *in vitro* bound edges between the 17 TFs with DAP-seq targets[8] (TFs in bold) and the 98 genes of this N-metabolism network and found more edges (529) in the TF-DNA binding assay for these 17 TFs (open grey circles), compared to those identified in root cells using TARGET for all 33 TFs. Red and blue shading indicate repression and induction, respectively, relative to the EV control. Source data are provided as a Source Data file.

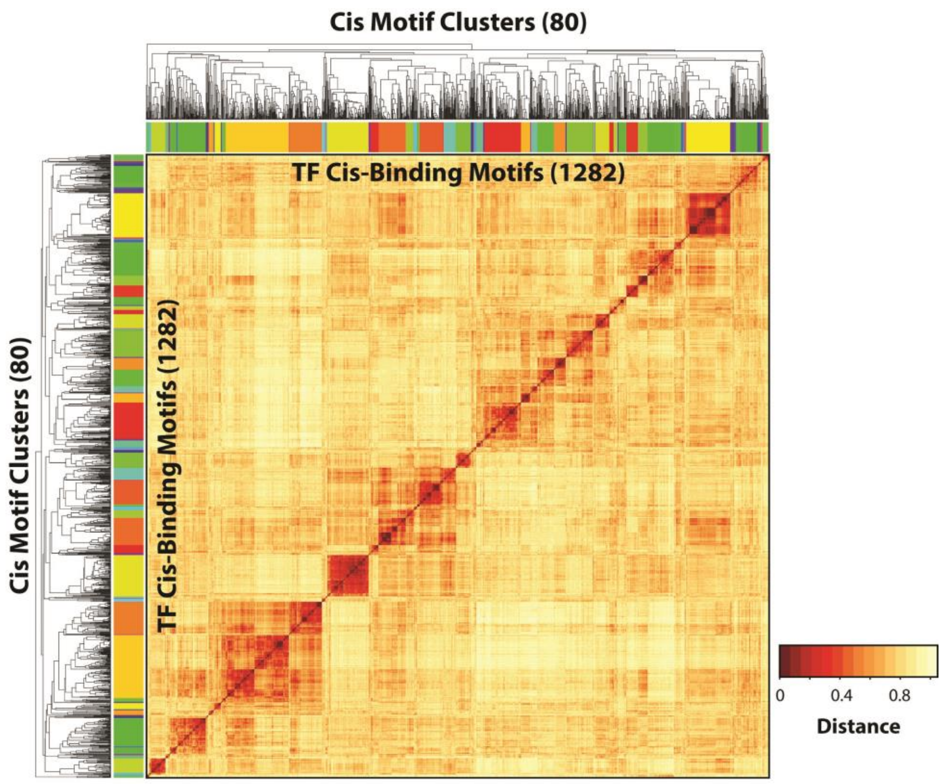

Supplementary Figure 10. Heatmap of cis-binding motif clustering for 1,282 Arabidopsis TF motifs into 80 groups

Heatmap of cis-binding motifs was generated by the RSAT matrix-clustering tool[12] based on the similarity (motif length normalized correlation (Ncor)) between 1,282 position weight matrices of cis-motifs for Arabidopsis TFs. Cis-binding motifs were collected from DAP-seq[8], Cis-BP[13], and the PBM of Zorilla *et al*[14]. Description of each consensus cis-motif and TF membership for each cluster can be found in Supplementary Data 8 and 9.

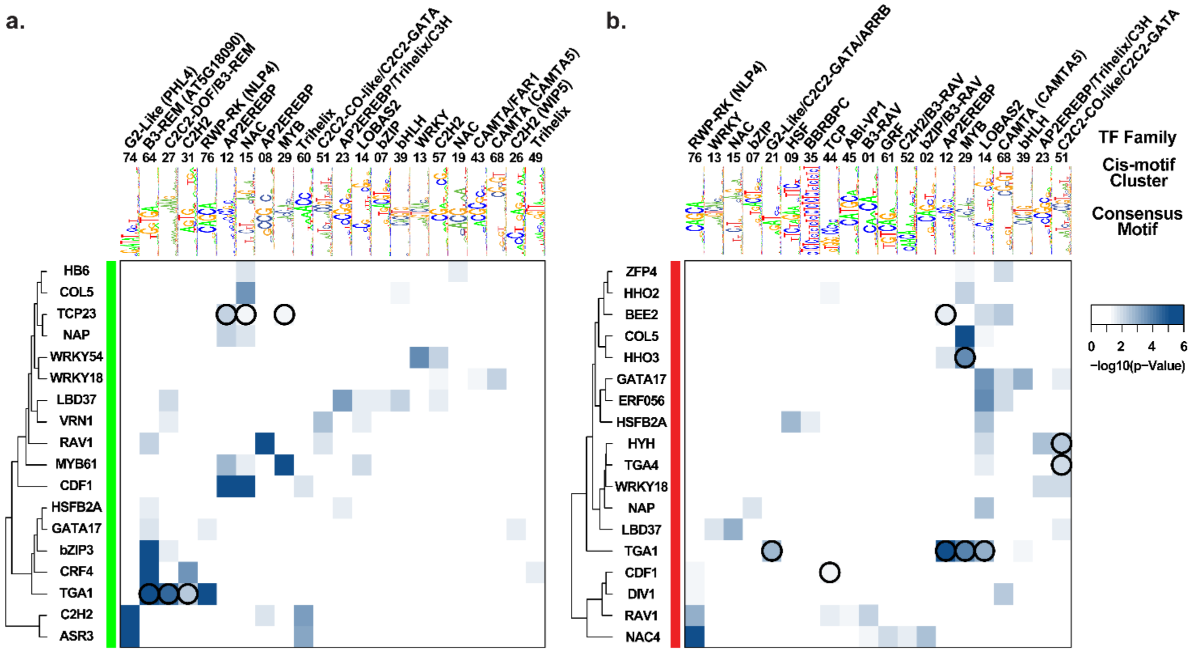

Supplementary Figure 11. The gene body of the targets of the 33 N-early response TFs are enriched in cis-regulatory elements for putative TF partners.

Heatmap of enriched consensus cis-motifs (CCMs) for the 80 cis-motif clusters (columns) in the gene body (5'UTR, CDS, introns, 3'UTR) of the (a) induced and (b) repressed direct regulated targets of the 33 N-early response TFs. The cis-motif logo, cluster number and family representation for each CCM is shown above. Instances where there is a validated interaction between the TF and another TF within a family represented by the enriched CCM cluster are marked by a black circle. Only TFs with enrichment of any of the 80 CCMs in the gene body are shown. Cis-binding motifs for Arabidopsis transcription factors were collected from DAP-seq[8], Cis-BP[13], and PBM[14]. Blue shading represents p-values calculated using Fisher's exact test and FDR corrected. Source data of Supplementary Figure 11a and 11b are provided as a Source Data file.

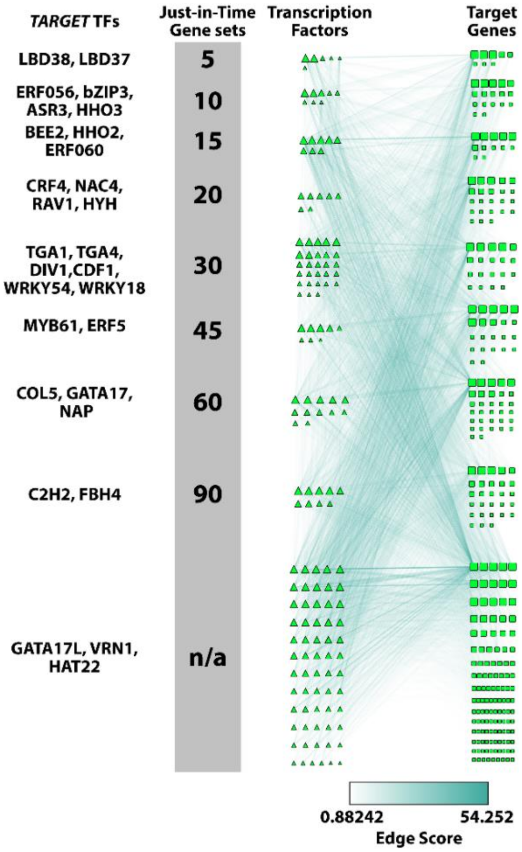

Supplementary Figure 12. A pruned network inferred using Dynamic Factor Graphs predicts high-confidence targets for 145 NxTime TFs

The root NxTime data from Varala *et al*.[6] was used to infer TF-target influence with a time-based machine learning approach, DFG[15]. Using genome-wide validated targets for 29 of the 33 TFs (Fig. 2) identified by the TARGET system in root cells, the inferred edges in the DFG inferred GRN were pruned to a precision threshold of 0.32, chosen based on AUPR analysis (Fig. 6 and Supplementary Table 2). This means that ~1/3 predicted TF-target edges are likely true. The resulting network is displayed in the context of the Just-in-Time bins for each TF (left) and NxTime TF-target genes (right)[6]. The size of the nodes is representative of the number of edges for each TF or target. The shading of the edges indicates the edge score from DFG.

Supplementary Table 1. Overlap of NxTime genes with targets regulated by the 33 TFs

	Overlap with 1458 root NxTime[6] genes	N-specificity p-value (root)	Overlap with 2020 shoot NxTime[6] genes	N-specificity p-value (shoot)
TARGET 15,460 validated targets	1288	1.55E-67	1785	5.65E-45

Supplementary Table 2. Overlap of *in planta* bound and direct regulated genes for HAT22 and HB6

	HAT22	HB6
in planta bound[10] (ChIP)	5902	7503
All direct regulated (TARGET)	2432	2623
Overlap all regulated/bound	1035	1270
p-value all regulated/bound (Fisher's exact test)	5.61E-56	3.09E-42
Induced direct regulated (TARGET)	1323	1130
Overlap induced regulated/bound	483	584
p-value induced regulated/bound (Fisher's exact test)	6.05E-11	1.65E-27
Repressed direct regulated (TARGET)	1109	1493
Overlap repressed regulated/bound	552	686
p-value repressed regulated/bound (Fisher's exact test)	4.47E-53	1.40E-15

Supplementary Table 3. Precision and Recall within the pruned network for TFs validated in TARGET

TF	Validated Edges	Predicted Edges	Validated Predictions	Precision	Recall	F-Score
All 18 TFs	2537	1031	330	32.0%	13.0%	0.18
GATA17L	96	68	29	42.6%	30.2%	0.35
ERF056	157	54	35	64.8%	22.3%	0.33
TGA4	67	31	13	41.9%	19.4%	0.27
C2H2	145	44	24	54.5%	16.6%	0.25
HYH	101	34	17	50.0%	16.8%	0.25
COL5	77	44	15	34.1%	19.5%	0.25
ERF060	83	71	19	26.8%	22.9%	0.25
MYB61	65	65	16	24.6%	24.6%	0.25
BEE2	35	207	29	14.0%	82.9%	0.24
FBH4	34	25	7	28.0%	20.6%	0.24
HHO2	116	39	18	46.2%	15.5%	0.23
RAV1	148	28	17	60.7%	11.5%	0.19
GATA17	158	21	15	71.4%	9.5%	0.17
WRKY54	45	27	6	22.2%	13.3%	0.17
WRKY18	33	16	4	25.0%	12.1%	0.16
ASR3	85	18	8	44.4%	9.4%	0.16
DIV1	121	24	10	41.7%	8.3%	0.14
CRF4	15	30	3	10.0%	20.0%	0.13
NAC4	67	43	7	16.3%	10.4%	0.13
HAT22	93	35	7	20.0%	7.5%	0.11
CDF1	85	11	5	45.5%	5.9%	0.10
bZIP3	141	20	8	40.0%	5.7%	0.10
VRN1	31	38	3	7.9%	9.7%	0.09
ERF5	50	3	2	66.7%	4.0%	0.08
HHO3	77	6	3	50.0%	3.9%	0.07
LBD38	87	6	3	50.0%	3.4%	0.06
TGA1	174	6	5	83.3%	2.9%	0.06
NAP	63	15	1	6.7%	1.6%	0.03
LBD37	88	2	1	50.0%	1.1%	0.02
Mean	-	-	-	39.3%	14.9%	0.17
95% Confidence Interval	-	-	-	32.0-46.5%	6.4-20.4%	0.14-0.20

Supplementary Table 4. Primers used for Gibson Assembly of 35S:TGA1

Primer Name	Sequence
35S Gibson Fwd	5′-AGCTATGACCATGATTACGCAGATTAGCCTTTTCAATTTCAGAAAGAATGCTAACC-3′
35S Gibson Rev	5′-TGTGTCGATGTCGAATTCATCGTGTTCTCTCCAAATGAAATGAACTTCCT-3′
TGA1 Gibson Fwd	5′-TTTCATTTGGAGAGAACACGATGAATTCGACATCGACACATTTTGTGCCA-3′
TGA1 Gibson Rev	5′-GATCGGGGAAATTCGAGCTCCTACGTTGGTTCACGATGTCGAGTTGC-3′
pGreen Gibson Fwd	5′-GACATCGTGAACCAACGTAGGAGCTCGAATTTCCCCGATCGTTCA-3′
pGreen Gibson Rev	5′-GAAATTGAAAAGGCTAATCTGCGTAATCATGGTCATAGCTGTTTCCT-3'

Supplementary References

1 Bargmann, B. O. *et al.* TARGET: a transient transformation system for genome-wide transcription factor target discovery. *Mol Plant* **6**, 978-980 (2013).
2 Kim, D. *et al.* TopHat2: accurate alignment of transcriptomes in the presence of insertions, deletions and gene fusions. *Genome Biol* **14**, R36 (2013).
3 Lawrence, M. *et al.* Software for computing and annotating genomic ranges. *PLoS Comput Biol* **9**, e1003118 (2013).
4 Love, M. I., Huber, W. & Anders, S. Moderated estimation of fold change and dispersion for RNA-seq data with DESeq2. *Genome Biol* **15**, 550 (2014).
5 Czechowski, T., Stitt, M., Altmann, T., Udvardi, M. K. & Scheible, W.-R. Genome-Wide Identification and Testing of Superior Reference Genes for Transcript Normalization in Arabidopsis. *Plant Physiol* **139**, 5-17 (2005).
6 Varala, K. *et al.* Temporal transcriptional logic of dynamic regulatory networks underlying nitrogen signaling and use in plants. *Proc Natl Acad Sci U S A* **115**, 6494-6499 (2018).
7 Murashige, T. & Skoog, F. A revised medium for rapid growth and bio assays with tobacco tissue cultures. *Physiol Plant* **15**, 473-497 (1962).
8 O'Malley, R. C. *et al.* Cistrome and Epicistrome Features Shape the Regulatory DNA Landscape. *Cell* **166**, 1598 (2016).
9 Birkenbihl, R. P., Kracher, B., Roccaro, M. & Somssich, I. E. Induced Genome-Wide Binding of Three Arabidopsis WRKY Transcription Factors during Early MAMP-Triggered Immunity. *Plant Cell* **29**, 20-38 (2017).
10 Song, L. *et al.* A transcription factor hierarchy defines an environmental stress response network. *Science* **354**, aag1550 (2016).
11 Gaudinier, A. *et al.* Transcriptional regulation of nitrogen-associated metabolism and growth. *Nature* **563**, 259-264 (2018).
12 Castro-Mondragon, J. A., Jaeger, S., Thieffry, D., Thomas-Chollier, M. & van Helden, J. RSAT matrix-clustering: dynamic exploration and redundancy reduction of transcription factor binding motif collections. *Nucleic Acids Res* **45** (2017).
13 Weirauch, M. T. *et al.* Determination and inference of eukaryotic transcription factor sequence specificity. *Cell* **158**, 1431-1443 (2014).
14 Franco-Zorrilla, J. M. *et al.* DNA-binding specificities of plant transcription factors and their potential to define target genes. *Proc Natl Acad Sci U S A* **111**, 2367-2372 (2014).
15 Mirowski, P. & LeCun, Y. Dynamic Factor Graphs for Time Series Modeling. *Lect Notes Artif Int* **5782**, 128-143 (2009).
16 Krouk, G., Lingeman, J., Colon, A. M., Coruzzi, G. & Shasha, D. Gene regulatory networks in plants: learning causality from time and perturbation. *Genome Biol* **14**, 123 (2013).
17 Krouk, G., Mirowski, P., LeCun, Y., Shasha, D. E. & Coruzzi, G. M. Predictive network modeling of the high-resolution dynamic plant transcriptome in response to nitrate. *Genome Biol* **11**, R123 (2010).
18 Bishop, C. M. *Pattern recognition and machine learning*. (Springer, 2006).
