# Supplementary material for: Network Walking charts transcriptional dynamics of nitrogen signaling by integrating validated and predicted genome-wide interactions
Source: Nat Commun. 2019 Apr 5;10:1569. doi: 10.1038/s41467-019-09522-1 (PMC6451032; doi:10.1038/s41467-019-09522-1)
Supplement: Supplementary file 2 — Reporting Summary [file 41467_2019_9522_MOESM2_ESM.pdf]

nature research

Corresponding author(s): Gloria Coruzzi

Last updated by author(s): 3/11/2019

Reporting Summary

Nature Research wishes to improve the reproducibility of the work that we publish. This form provides structure for consistency and transparency in reporting. For further information on Nature Research policies, see Authors & Referees and the Editorial Policy Checklist.

Statistics

For all statistical analyses, confirm that the following items are present in the figure legend, table legend, main text, or Methods section.

n/a	Confirmed	
☐	☒	The exact sample size (*n*) for each experimental group/condition, given as a discrete number and unit of measurement
☐	☒	A statement on whether measurements were taken from distinct samples or whether the same sample was measured repeatedly
☐	☒	The statistical test(s) used AND whether they are one- or two-sided<br>*Only common tests should be described solely by name; describe more complex techniques in the Methods section.*
☐	☒	A description of all covariates tested
☐	☒	A description of any assumptions or corrections, such as tests of normality and adjustment for multiple comparisons
☒	☐	A full description of the statistical parameters including central tendency (e.g. means) or other basic estimates (e.g. regression coefficient) AND variation (e.g. standard deviation) or associated estimates of uncertainty (e.g. confidence intervals)
☐	☒	For null hypothesis testing, the test statistic (e.g. F, t, r) with confidence intervals, effect sizes, degrees of freedom and P value noted<br>*Give P values as exact values whenever suitable.*
☒	☐	For Bayesian analysis, information on the choice of priors and Markov chain Monte Carlo settings
☒	☐	For hierarchical and complex designs, identification of the appropriate level for tests and full reporting of outcomes
☒	☐	Estimates of effect sizes (e.g. Cohen's *d*, Pearson's *r*), indicating how they were calculated

Our web collection on statistics for biologists contains articles on many of the points above.

Software and code

Policy information about availability of computer code

Data collection	no software was used for data collection
Data analysis	The following R packages were used:GenomicAlignments_1.18.0 and GenomicFeatures_1.34.1 to estimate gene expression, DESeq2_1.22.1 to identify differentially expressed genes, gplots_3.0.1 and ggplot2_3.1.0 for data visualization. AgriGO v2 was used for GO term analysis (http://systemsbiology.cau.edu.cn/agriGOv2/). Cytoscape v3.4.0 was used to create networks. The AME and FIMO tools from the MEME Suite v4.11.4 were used for motif analysis and enrichment (http://http://meme-suite.org). The matrix clustering tool within the Regulatory Sequence Analysis Tools project (http://rsat.sb-roscoff.fr/) was used to cluster Arabidopsis cis-motifs.

For manuscripts utilizing custom algorithms or software that are central to the research but not yet described in published literature, software must be made available to editors/reviewers. We strongly encourage code deposition in a community repository (e.g. GitHub). See the Nature Research guidelines for submitting code & software for further information.

Data

Policy information about availability of data

All manuscripts must include a data availability statement. This statement should provide the following information, where applicable:

- Accession codes, unique identifiers, or web links for publicly available datasets
- A list of figures that have associated raw data
- A description of any restrictions on data availability

All raw sequencing data from this project have been deposited in the Gene Expression Omnibus (GEO) database accession GSE117857 [https://www.ncbi.nlm.nih.gov/geo/query/acc.cgi?acc=GSE117857] and GSE128209 [https://www.ncbi.nlm.nih.gov/geo/query/acc.cgi?acc=GSE128209]. Data supporting the findings of this work are available within the paper and its Supplementary Information files. A reporting summary for this Article is available as a Supplementary Information file. The datasets generated and analyzed during the current study are available from the corresponding author on reasonable request. The source data underlying Figures 3a, 3b, 5a, 5b, and 6, as well as Supplementary Figures 2, 4b-d, 5, 6, 8, 9, 11a, and 11b are provided as a Source Data file.

Field-specific reporting

Please select the one below that is the best fit for your research. If you are not sure, read the appropriate sections before making your selection.

☒ Life sciences ☐ Behavioural & social sciences ☐ Ecological, evolutionary & environmental sciences

For a reference copy of the document with all sections, see nature.com/documents/nr-reporting-summary-flat.pdf

Life sciences study design

All studies must disclose on these points even when the disclosure is negative.

Sample size	Sample sizes were chosen based on previous experience in testing for gene expression responses to N exposure using the TARGET system
Data exclusions	No data was excluded from analysis.
Replication	All experimental parameters were tested in biological triplicate.
Randomization	Empty vector control was included on each day TARGET experiments were performed and the DESeq2 model accounted for the batch effect
Blinding	Blinding was not relevant to this study.

Reporting for specific materials, systems and methods

We require information from authors about some types of materials, experimental systems and methods used in many studies. Here, indicate whether each material, system or method listed is relevant to your study. If you are not sure if a list item applies to your research, read the appropriate section before selecting a response.

Materials & experimental systems

n/a	Involved in the study	
☒	☐	Antibodies
☒	☐	Eukaryotic cell lines
☒	☐	Palaeontology
☒	☐	Animals and other organisms
☒	☐	Human research participants
☒	☐	Clinical data

Methods

n/a	Involved in the study	
☒	☐	ChIP-seq
☒	☐	Flow cytometry
☒	☐	MRI-based neuroimaging
