# Supplementary material for: Network Walking charts transcriptional dynamics of nitrogen signaling by integrating validated and predicted genome-wide interactions
Source: Nat Commun. 2019 Apr 5;10:1569. doi: 10.1038/s41467-019-09522-1 (PMC6451032; doi:10.1038/s41467-019-09522-1)
Supplement: Supplementary file 3 — Description of Additional Supplementary Files [file 41467_2019_9522_MOESM3_ESM.docx]

**Description of Additional Supplementary Files**

File Name: Supplementary Data 1

Description: Genes that respond to cycloheximide treatment of root protoplasts with a fold-change greater than 5

File Name: Supplementary Data 2

Description: Differentially expressed gene targets of 33 nitrogen-early response identified in TF-transfected root protoplasts

File Name: Supplementary Data 3

Description: Overlap between *in vitro* targets identified by DAP-seq and direct TFregulated targets in root cells from this study

File Name: Supplementary Data 4

Description: Differentially expressed targets of TGA1 (35S:TGA1 vs Col-0) identified in whole roots

File Name: Supplementary Data 5
Description: Gene Ontology terms enriched in the cumulative direct regulated targets of 33 N-early response TFs identified in root cells using *TARGET*

File Name: Supplementary Data 6

Description: Enrichment of known TF cis-binding motifs in promoter and gene features of direct regulated TF targets identified in root cells using *TARGET*

File Name: Supplementary Data 7

Description: Enrichment of known TF cis-binding motifs in 500bp promoter of direct regulated TF targets identified in root cells after DHS filtering

File Name: Supplementary Data 8

Description: Summary of the 80 cis-binding motif clusters of Arabidopsis generated by the RSAT matrix-clustering tool

File Name: Supplementary Data 9
Description: Arabidopsis TF cis-binding motifs enriched in each of the 80 cis-clusters

File Name: Supplementary Data 10

Description: Time-inferred DFG network of N-response in whole roots pruned for precision using TF-targets validated in root cells using *TARGET*

File Name: Supplementary Data 11

Description: Differentially expressed targets of CRF4 identified using inducible expression *in planta* from Varala et al. PNAS 2018

File Name: Supplementary Data 12
Description: Established *in planta* roles of the 33 nitrogen-early response TFs from the literature
